# Temperature-Immune High-Entropy Alloy Flexible Strain Sensor on Electrospinning Nanofibrous Membrane

**DOI:** 10.1007/s40820-025-02033-3

**Published:** 2026-01-12

**Authors:** Wenxin Li, Xianruo Du, Yisheng Zhong, Ruixin Chen, Yuyang Wang, Huatan Chen, Huangping Yan, Yifang Liu, Chentao Zhang, Gaofeng Zheng

**Affiliations:** 1 College of Physical Science and Technology, Xiamen University, Xiamen, 361005 People’s Republic of China; 2 Pen-Tung Sah Institute of Micro-Nano Science and Technology, Xiamen University, Xiamen, 361005 People’s Republic of China; 3 State Key Laboratory of Flexible Electronics Technology, Tsinghua University, Beijing, 100084 People’s Republic of China; 4 School of Mechanical and Automotive Engineering, Xiamen University of Technology, Xiamen, 361024 People’s Republic of China

**Keywords:** High-entropy alloy nanofibers, Flexible strain sensors, Electrospinning, Temperature immunity

## Abstract

Temperature stability is essential for the precision of flexible sensors. However, constrained by the composite principle of heterogeneous materials, the existing self-compensating methods encounter substantial challenges. To tackle this, high-entropy alloy nanofibers were utilized to construct a flexible strain sensor with inherent temperature stability. This approach leverages the electrohydrodynamic direct writing; a precursor conductive network was established through the electrospinning of a high-entropy alloy acetate and polyvinylidene difluoride solution blend. Subsequently, annealing treatment facilitated metallization, resulting in the synergistic preservation of polymer stretchability and the low temperature coefficient of resistance properties of high-entropy alloys inside the nanofibers. The test results demonstrate that the high-entropy alloys flexible strain sensor exhibits a remarkably low temperature coefficient of resistance (45.59 ppm K^−1^) across the range of − 10 to 70 °C, a sensitivity coefficient GF of 1.12 with a 50% strain range, and a response time of 310 ms. After 6000 stretching cycles, no baseline drift or failure occurred, indicating excellent cyclic stability. Furthermore, the outstanding temperature stability of the sensor was validated through wearable application and robotic hands strain sensing conducted under varied environment temperatures. This work provides a viable design pathway for developing flexible sensors with an inherently low temperature coefficient of resistance.

**Supplementary Information:**

The online version contains supplementary material available at 10.1007/s40820-025-02033-3.

## Highlights

- High-entropy alloy fiber was fabricated via electrohydrodynamic direct writing and subsequently metallized at the nanoscale to form uniform high-entropy alloy lattices within polymer nanofibers.
- The metallized temperature-immune strain sensor exhibits low temperature coefficient of resistance (45.59 ppm K^-1^) and excellent cyclic stability (6000 cycles), enabling reliable strain measurements across a wide temperature range.
- Wearable human joint monitoring and robotic grasping tests demonstrate the sensor’s high reliability and accurate response under complex thermal environments.

**Supplementary Information:**

The online version contains supplementary material available at 10.1007/s40820-025-02033-3.

## Graphical abstract

**Figure:**
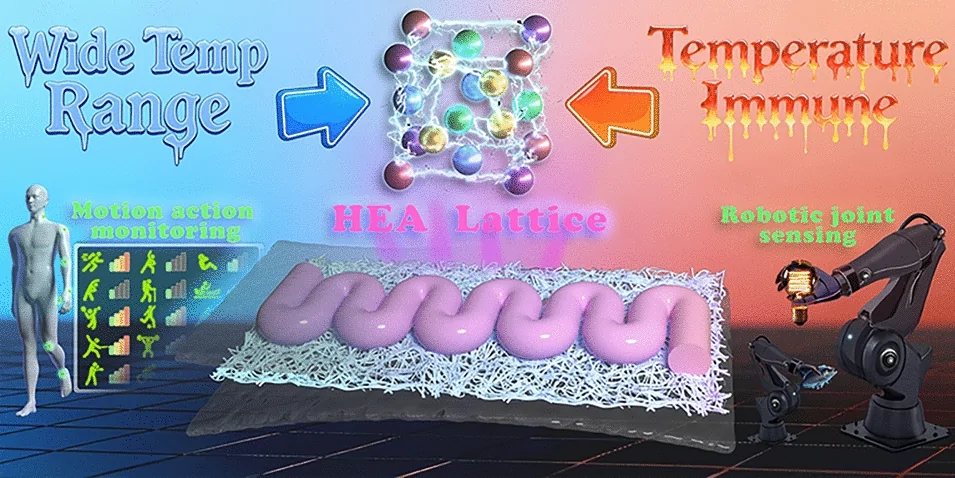

## Introduction

Flexible sensors are essential in contemporary sensing systems. With the swift progress of next-generation technological devices [1–3], flexible sensors are extensively utilized in various sensing contexts [4–9]. However, unlike rigid devices equipped with robust encapsulations and efficient temperature management, flexible sensors feature compact dimensions and are fabricated on flexible substrates [10–14]. These characteristics hinder the effective isolation of external thermal disturbances, rendering flexible sensors highly susceptible to temperature fluctuations in the operating environment [15]. Such fluctuations pose considerable challenges to signal accuracy and stability. This deficiency is mostly ascribed to the performance variability of flexible sensing devices induced by temperature fluctuations [16–19]. Signal crosstalk between temperature and the goal measurement, evident as baseline drift [20, 21], noise amplification [22], and dynamic measurement errors [23], has emerged as a significant impediment to the extensive deployment of multiple flexible sensors [24, 25]. Therefore, addressing temperature-induced signal disruptions is crucial for maintaining the stable performance of flexible sensors in variable thermal environments.

To address the performance challenges induced by temperature fluctuations, materials exhibiting positive temperature coefficients (PTC) and negative temperature coefficients (NTC) have been extensively researched to produce sensitive components in flexible sensors [26, 27]. Zhu et al. combined PTC carbon nanotubes (CNT) with NTC graphene and carbon black (CB) to produce pressure- and strain-sensitive layers with an overall temperature coefficient of resistance (TCR) near zero [28]. Similar PTC–NTC hybrid architectures have been reported to achieve near-zero or compensated TCR through composition tuning and percolation balancing [29–32]. Gao et al. achieved continuous tuning of CNT materials’ TCR from negative to positive by adjusting the annealing temperature, which is attributed to the alteration of the ratio between physical contacts and covalent bonds among CNTs [33, 34]. Moreover, metal nanoparticles not only exhibit exceptional electrical conductivity but also reveal typical metallic PTC properties. Choi et al. developed a thermally responsive complementary conductive network by integrating nano-metallic materials with NTC constituents, wherein Ag nanoparticles (Ag NPs) and Ag nanowires (Ag NWs) are combined with negative temperature coefficient materials to establish a complementary pathway [35]. Such complementary conductive networks, which utilize metallic PTC elements together with NTC constituents, have been observed to exhibit extremely low TCR across broad temperature ranges [36–40]. Based on the material optimization strategy, researchers have commenced exploring the concept of structural-material synergistic compensation. Yuan et al. employed the elastic substrates with thermal expansion properties, such as polydimethylsiloxane (PDMS), to mitigate the adverse temperature response of NTC materials via multilayer composites [41–43]. This approach results in flexible sensors with a low TCR and self-compensating attributes. Although the previously proposed strategies have shown efficacy in mitigating temperature-induced signal disturbances, composite compensation systems still face significant limitations. The constituent materials often exhibit inconsistent thermal responses, and some are susceptible to oxidation or chemical degradation. These factors may compromise the internal cooperative stability of the system. If the compensating function of a single component fails, maintaining the overall compensation effect becomes difficult, ultimately jeopardizing the system’s long-term reliability.

**Fig. 1:**
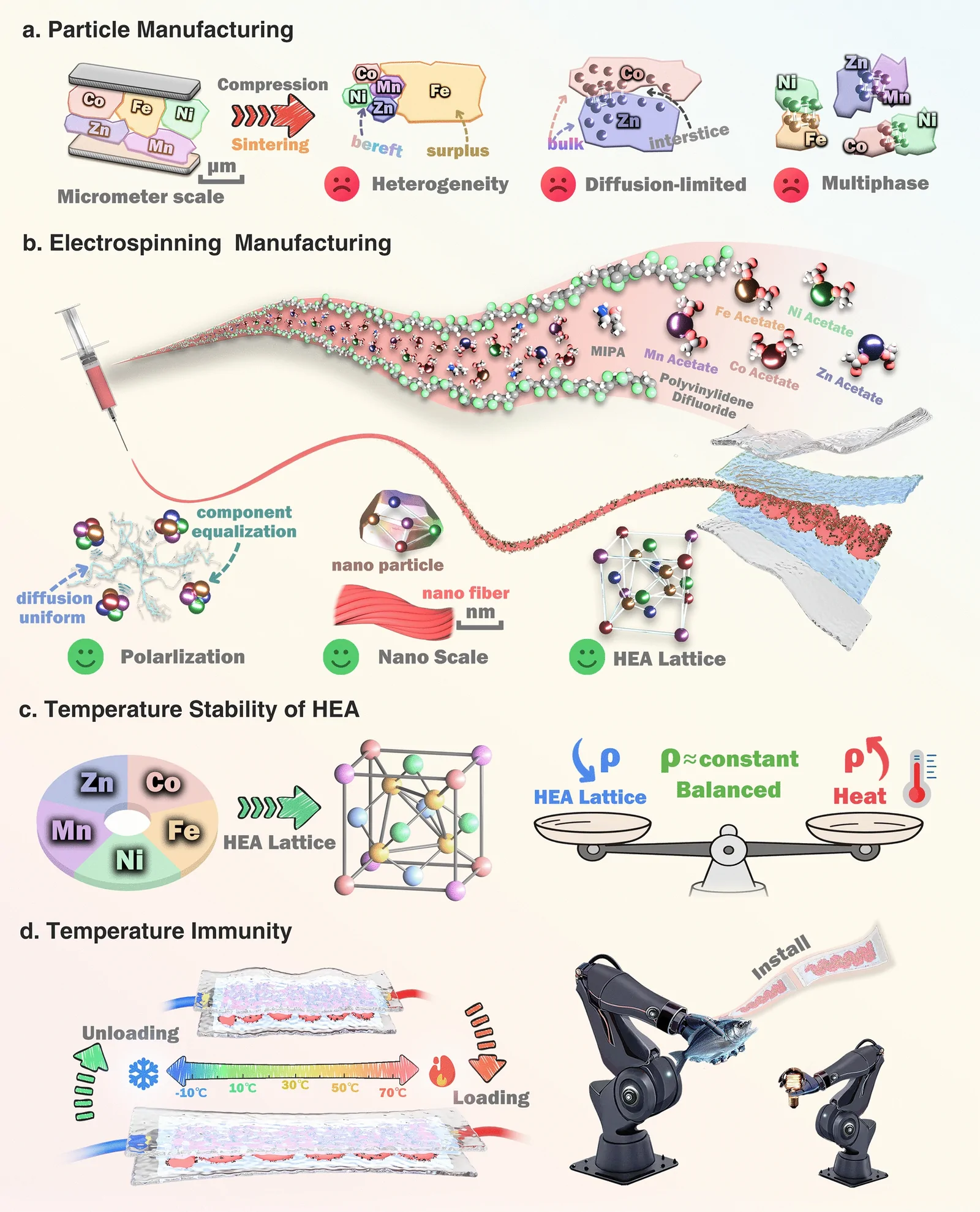
Schematic diagram of the temperature-immune high-entropy alloy strain sensor. **a** Insufficient particle fabrication and high-entropy alloy synthesis. **b** Electrospinning to fabricate nanofibers of high-entropy alloys. **c** Temperature stability and TCR balance mechanism of high-entropy alloys. **d** Application of temperature-immune strain sensors

Given the limitations of the material composite self-compensation approach in maintaining thermal stability, developing sensor components with intrinsic superior temperature stability plays a pivotal role in ensuring the reliable operation of flexible sensors in situations with fluctuating temperatures. High-entropy alloys (HEAs) have attracted considerable attention from researchers due to their unique compositional complexity and severe lattice distortion [44–46]. Shafeie et al. investigated the electron–phonon scattering mechanisms in a class of HEA constituents exhibiting exceptionally low TCR [47]. Subsequent studies have further confirmed that such alloy materials can underscore their potential in the development of thermally stable electronic devices [48]. Subsequently, Benrazzouq et al. introduced aluminum into an established HEA system, inducing pronounced lattice distortion and modifying the internal scattering mechanisms [49]. This structural modification suppressed elastic electron–phonon scattering, thereby reducing the sensitivity of electrical resistance to temperature fluctuations [50]. As a result, the TCR of the HEA films could be continuously tuned from negative to positive values at extremely low absolute levels, offering enhanced design flexibility for thermally stable electronic materials. However, to fully exploit the potential of this class of materials in flexible sensors, challenges in achieving compositional homogeneity and phase stability during fabrication must be addressed. Existing studies have predominantly employed solid-state routes involving powder compaction and sintering. Yet the limited atomic diffusion efficiency of micron-scale particles under solid-state conditions poses a challenge to achieving sufficient multicomponent interdiffusion and compositional uniformity between particles [51–53] (Fig. 1a). This leads to elemental segregation and multiphase precipitation, thereby constraining the formation of high-entropy alloy lattices [54, 55]. To overcome these limitations, electrospun nanofiber fabrication has emerged as a promising strategy. Leveraging the atomic-level homogeneity of solution precursors and the confinement effects at the nanoscale, this approach markedly enhances atomic diffusion efficiency and effectively suppresses segregation tendencies [56–58]. During electrospinning, the applied electric field drives fiber formation but also induces polarization alignment of ions and atoms within the precursor system, thereby significantly promoting uniform multi-component distribution and cooperative diffusion at the nanoscale [56, 59, 60]. As a result, high-entropy alloy lattices with uniform composition and stable phase structures can be formed within the nanofibers, providing a solid foundation for maintaining stable sensor performance under thermal perturbations and ensuring consistent electrical characteristics of the device (Fig. 1b, c).

Herein, to characterize the low TCR of high-entropy alloys on flexible fibers and utilize them as the sensitive layer in strain sensors. A FeCoNiMnZn/PVDF flexible strain sensor was fabricated using electrohydrodynamic direct-write technology. By metallization annealing of the HEA components within the polymer nanofiber matrix, the inherent atomic configurational disorder was exploited to induce pronounced lattice distortion, thereby enhancing structural stability and establishing robust, continuous electron transport pathways. The significant lattice distortion and chemical disorder effectively weaken electron–phonon coupling, reducing the dependence of electron transport channels on temperature variations and thus enabling nearly temperature-insensitive conduction. In addition, the nanoscale uniform metallization structure achieved through electrohydrodynamic direct writing ensures a highly homogeneous distribution of alloying elements within the fibers, forming a continuous and stable conductive network. This microstructural optimization stabilizes resistance against temperature-induced fluctuations, resulting in a reduced TCR. It prevents resistance drift caused by interfacial instability during thermal cycling, ultimately enabling reliable temperature-insensitive strain detection (Fig. 1d). Furthermore, the sensor was integrated into wearable joint strain monitoring and robotic grasping systems, underscoring its excellent thermal stability under varying temperature conditions. Through metallization of high-entropy alloy components within a nanofiber matrix, a flexible strain sensor that combines intrinsic temperature stability with high sensitivity, rapid response, and long-term operational reliability is presented. This study provides an effective strategy for stable sensing in thermally dynamic environments and expands the application boundaries of flexible electronics in complex thermal conditions.

## Experimental Section

### Materials

The materials used were polyvinylidene difluoride (PVDF, Mw = 600,000, Arkema S.A.), manganese acetate tetrahydrate [(CH_3_COO)_2_Mn, purity 99.0%, Sinopharm Chemical Reagent Co., Ltd.], nickel acetate tetrahydrate [(CH_3_COO)_2_Ni, purity 98.0%, Sinopharm Chemical Reagent Co., Ltd.], cobalt acetate tetrahydrate [(CH_3_COO)_2_Co, purity 98.0%, Sinopharm Chemical Reagent Co., Ltd.], zinc acetate dihydrate [(CH_3_COO)_2_Zn, purity 99.0%, Xilong Scientific Co., Ltd.], iron acetate tetrahydrate [(CH_3_COO)_2_Fe(OH), purity 99.0%, Shanghai Acmec Biochemical Technology Co., Ltd.], iso-propanol amine (C_3_H_9_NO, purity 99.0%, Shandong Yousuo Chemical Technology Co., Ltd.), N,N-dimethylformamide (DMF, purity 99.9%, Shanghai Acmec Biochemical Technology Co., Ltd.), and polyurethane film (PU, Jiangsu Hongsheng Bioengineering Co., Ltd.).

### Preparation of PVDF/DMF and HEA-Salt Precursor Solution

A PVDF/DMF solution was prepared using 13 wt% PVDF content. One gram of PVDF was dissolved in 6.7 g of DMF solution; the mixture was sealed and stirred magnetically at 25 °C for 48 h. A HEA-salt precursor solution containing 8.2 wt% metal salt, 8.3 wt% PVDF and 15 wt% iso-propanol amine was prepared. One gram of PVDF was dissolved in 8.2 g DMF solution; the mixture was sealed and stirred magnetically at 25 °C for 48 h. After dissolving completely, 0.0008 mol manganese acetate, 0.0008 mol nickel acetate, 0.0008 mol cobalt acetate, 0.0008 mol iron acetate, 0.0009 mol zinc acetate and 1.8 g isopropanol amine were added successively. The mixture was sealed and stirred magnetically at 25 °C for 24 h. The schematic diagram of the solution configuration can be found in Figs. S1 and S2.

### Electrospinning and Electrohydrodynamic Direct Writing System

The solution was stored in a 1-mL syringe (Jiangsu Zhiyu Medical Equipment Co., Ltd., China) and delivered by a precision syringe pump (Pump 11 Pico Plus Elite, Harvard Instruments, USA) to a stainless-steel needle with an outer diameter of 0.23 mm and an inner diameter of 0.08 mm. The needle was connected to the positive terminal of a high-voltage power supply (DW-SA403-1ACE5, Tianjin Dongwen High Voltage Power Supply Co., Ltd., China). An electrospinning apparatus (QZNT-E01-01, Foshan Qingzi Precision Measurement & Control Technology Co., Ltd., China) allowing the fibers to accumulate layer by layer on the surface. The electrohydrodynamic direct writing was driven by a motion platform (POT-G-MOT-F09-06, Jiangxi Liansheng Precision Optical Platform Co., Ltd., China) to deposit nanofibers with a designed structure. The experimental parameters for the electrospinning substrate and the electrohydrodynamic direct writing of high-entropy alloy nanofibers are summarized in Tables S1 and S2.

### Microstructural and Property Measurements

The HEA/PVDF fiber morphology was determined by field emission scanning electron microscopy (SUPRA55 SAPPHIRE, Carl Zeiss AG, Germany). Raman spectra of the samples were recorded by confocal Raman microscope (lDSPeC ARCTlC, Beijing Pole Spectrometer Technology Co., LTD., China). Selected area electron diffraction (SEAD) was obtained by transmission electron microscopy (FEI Company, USA). X-ray diffraction (XRD) spectra of the samples were measured using an XRD-7000 instrument (Shimadzu Corporation, Japan). Fourier transform infrared (FTIR) spectra of the samples were recorded on a MICOET iS10 instrument (Thermo Fisher Scientific, USA). Mechanical tensile properties were measured using a tensile testing machine (ZQ-990B, Dongguan Zhiqu Precision Instrument Co., LTD., China). The LCR digital bridge (TH2830, Changzhou Tonghui Electronics Co., Ltd., China) tested the electrical performance of the sensor. The samples were sintered by split-type micro-dust graphite heating plate (IEH54-1, Lichen Technology Co., Ltd., China). The infrared thermal image was obtained using a Professional Thermal Imager (UTi320E, Uni-Trend Technology Co., Ltd., China). The thermogravimetric analysis (TGA) of the samples was obtained using a thermogravimetric analyzer (Netzsch TGA 209 F1, NETZSCH, Germany). The thermomechanical analysis (TMA) data of the samples were obtained using a thermomechanical analyzer (Netzsch TMA 402 F3, NETZSCH, Germany).

## Results and Discussion

### Fabrication of Sensor

**Fig. 2:**
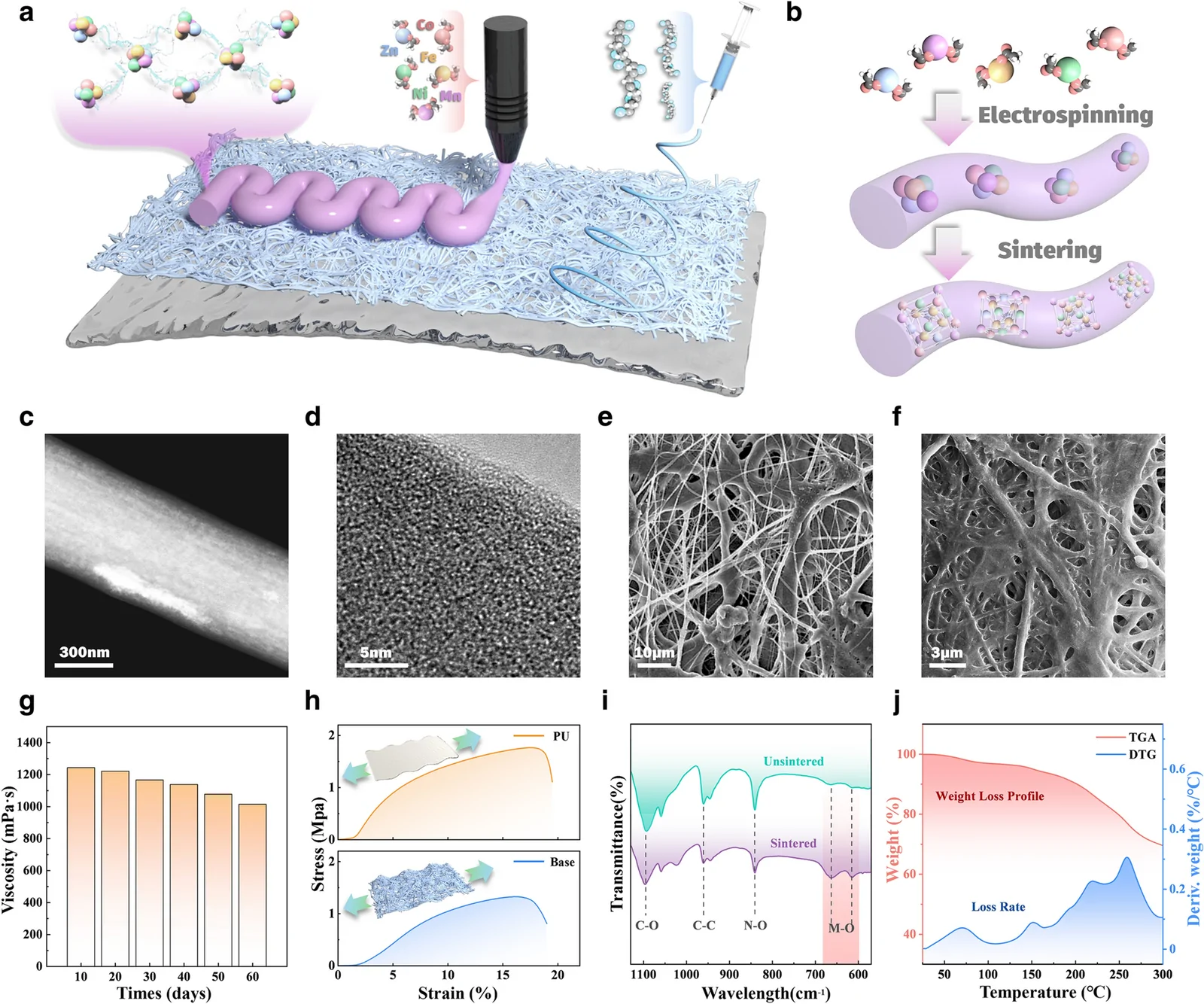
Schematic diagram of the sensor preparation process. **a** Schematic diagram of high-entropy alloy fibers using electrohydrodynamic direct writing and electrospinning for substrate preparation. **b** High-entropy alloy components form a lattice within the nanofibers. **c**, **d** STEM images of high-entropy alloy fibers. **e, f** STEM images of the basal fibers. **g** Viscosity of the high-entropy alloy solution changes over time. **h** Strain–stress diagrams of the electrospun substrate and the PU membrane. **i** Comparison of FTIR tests of high-entropy alloy fibers before and after sintering. **j** Thermogravimetric analysis curve of high-entropy alloy fibers

Electrospinning was employed to fabricate flexible strain sensors. A homogeneous solution suitable for electrospinning was first prepared by dissolving PVDF in DMF, followed by continuous stirring for 48 h. To obtain the HEA-containing precursor solution, metal acetates of the HEA components and iso-propanol amine were added to the PVDF/DMF solution, and the mixture was stirred for an additional 12 h. The change in viscosity of the high-entropy alloy solution over time is shown in Fig. 2g. During electrospinning, a high-voltage electric field between the needle and the collector generated a stable jet, leading to continuous fiber deposition on the substrate. Electrospinning the PVDF/DMF solution yielded a shear-collected PVDF nanofiber membrane that served as the flexible base layer. The working principle of the electrospinning platform is illustrated in Fig. S3.

Using electrohydrodynamic direct writing, a reciprocating pattern was printed onto the PVDF nanofiber membrane substrate (Fig. 2a). The collaborative effect of multiple components of the electrohydrodynamic direct writing platform is illustrated in Figs. S4 and S5. The details of the STEM images of the substrate and the HEA fibers are shown in Fig. 2c–f, which presents the fiber morphology of the substrate and the grains within the fibers of the high-entropy alloy. During the electrospinning of the HEA solution, the electric field-induced polarization enhanced the diffusion efficiency of metal atoms within the nanofibers and effectively suppressed compositional segregation, thereby ensuring overall compositional homogeneity and promoting the subsequent formation of a stable high-entropy alloy lattice. Compared with alloys prepared by the laser particle sintering method in other studies (Fig. S6b), compositional fluctuations were observed at the 5-µm scale. These alloys also exhibit clearly defined grain boundaries, indicating the presence of segregation and agglomeration. In contrast, the high-entropy alloy nanofibers fabricated in this work showed a markedly different microstructural feature. As presented in Fig. S6a, the compositional distribution was examined at a finer 200-nm scale using EDS elemental mapping. The analysis confirms that, after the electric-field-induced polarization treatment, the five metallic elements were uniformly distributed. No evidence of compositional segregation or elemental clustering was detected.

The resulting double-sided structure was subjected to thermal treatment at 300 °C for 1 h, which enabled characterization of the HEA precursor fibers while preserving their fibrous morphology. In Fig. 2b, during the electrospinning process, the applied electric field drove fiber formation and induced polarization of the metal atoms in the precursors. Under the influence of the electric field, different metal atoms in the high-entropy alloy underwent electronic displacement polarization and dipole orientation polarization, ultimately achieving uniform distribution within the fibers. This nanoscale homogeneity within the fibers provided favorable conditions for the migration and bonding of metal atoms during subsequent sintering, facilitating the formation of compositionally uniform and structurally stable high-entropy alloy lattices. The lattice fringes in local regions, together with measurements of the interplanar spacing, are presented in Fig. S7. It can be observed that within the continuous lattice of the high-entropy alloy, multiple scales of interplanar spacing exist. In certain areas, long-range ordered parallel lattice fringes failed to form due to the pronounced lattice distortion effect inherent to high-entropy alloys. This heterogeneity and diversity at the lattice scale arose from the uniform mixing of multiple principal-element atoms, which jointly formed a solid-solution phase and induced strong lattice distortion. The FTIR spectra and weight loss before and after sintering of the HEA sample are shown in Fig. 2i, j. The thermogravimetric analysis of the electrospun PVDF membrane is shown in Fig. S8. As the sintering process was completed, thermogravimetric analysis recorded the changes in mass and the mass change rate. Additionally, at wavelengths of 615 and 663 cm^−1^, FTIR revealed peaks characteristic of the bonds between metal and oxygen atoms. Finally, conductive silver paste was applied to both ends of the sensor to connect external wires, and a polyurethane (PU) film was laminated onto the outer surface as a protective layer, completing the fabrication of the strain sensor device. The stress–strain diagrams of the PU membrane and the electrospun substrate are shown in Fig. 2h. The closely matched mechanical strength ensured synergistic deformation during strain detection, thus guaranteeing the accuracy and consistency of strain transfer.

### Materials and Structural Characterization of Strain Sensor

**Fig. 3:**
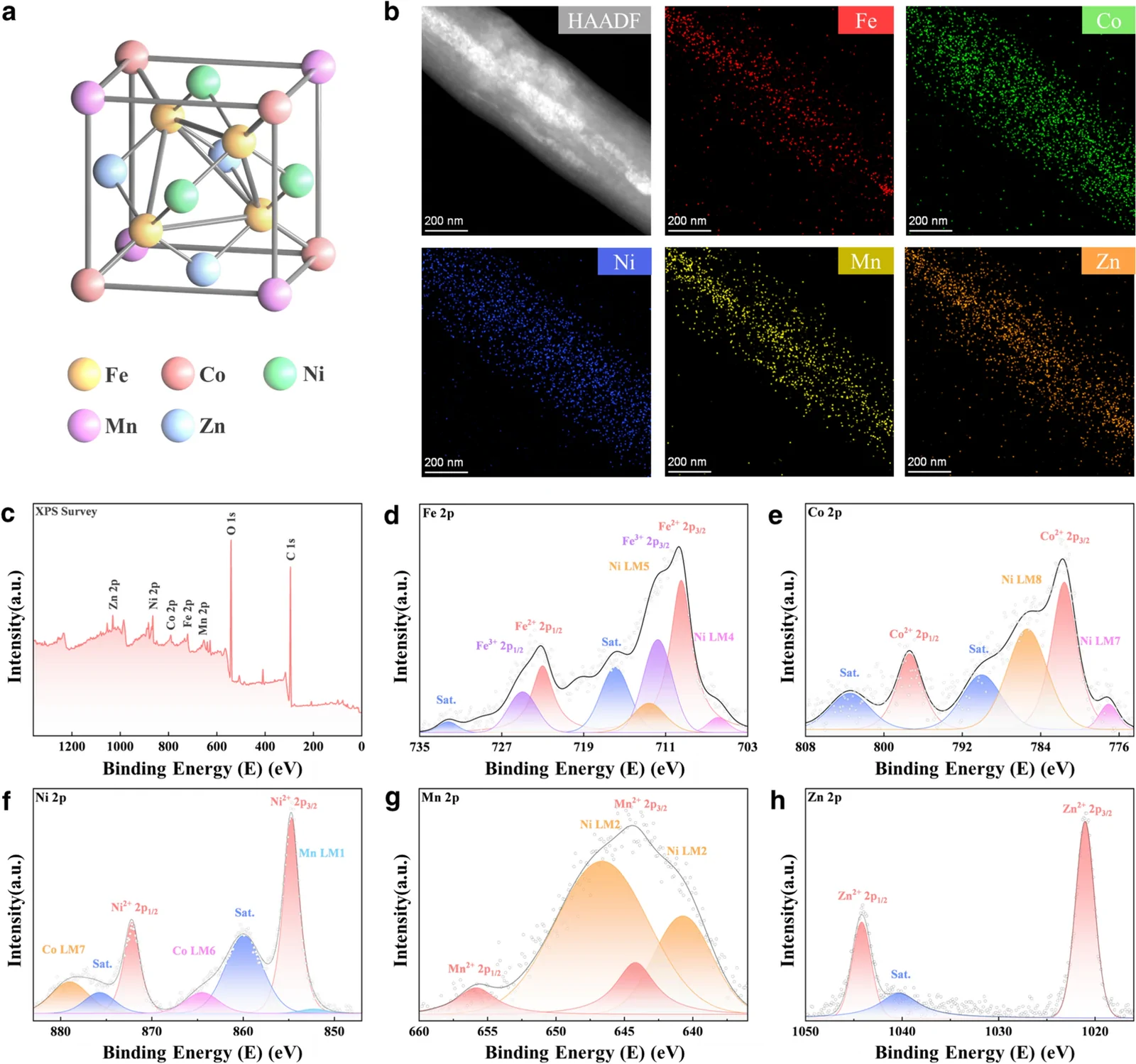
Surface chemical states, morphological and structural characterization of FeCoNiMnZn/PVDF fiber characterized by scanning transmission electron microscopy (STEM) and X-ray photoelectron spectroscopy (XPS). **a** Lattice structure of HEA. **b** The corresponding STEM-EDS mapping images of Fe, Co, Ni, Mn and Zn. The high-resolution XPS spectra of **c** XPS survey, **d** Fe 2*p*, **e** Co 2*p*, **f** Ni 2*p*, **g** Mn 2*p* and **h** Zn 2*p*

To explore the surface of high-entropy alloy fiber with distorted lattice (Fig. 3a), the morphology and element distribution of single fibers were explored by HAADF-STEM and STEM-EDS in Fig. 3b, which shows the homogeneous distribution of elements Fe, Co, Ni, Mn, and Zn in a single FeCoNiMnZn/PVDF fiber. X-ray photoelectron spectroscopy (XPS) was applied to explore the electronic effects and surface composition of the HEA fiber manufactured by FeCoNiMnZn/PVDF. The XPS survey spectrum presented in Fig. 3c, d indicates the double peaks of Fe^2+^ and Fe^3+^. The binding energy (BE) at 709.6, 711.8, 723.0, and 724.9 eV is ascribed to Fe^2+^ 2*p*_3/2_, Fe^3+^ 2*p*_3/2_, Fe^2+^ 2*p*_1/2_, and Fe^3+^ 2*p*_1/2_, respectively. Additionally, two satellite peaks (marked as “Sat.”) are observed at 715.9 eV and 732.3 eV. The pre-peak before the main Fe^3+^ 2*p*_3/2_ peak is attributed to the Ni LM8 Auger lines, and the post-peak after the main Fe^2+^ 2*p*_3/2_ peak is attributed to the Auger lines of Ni LM4. For the spectrum of Co 2*p* (Fig. 3e), the main peaks appear in the BE of 781.6 and 791.4 eV, which, respectively, belong to Co^2+^ 2*p*_3/2_ and Co^2+^ 2*p*_1/2_. Two satellite peaks are located at 790.0 and 803.5 eV. The Ni LM8 Auger line appears as a pre-peak before the Co^2^⁺ 2*p*_3/2_ main peak, and the Ni LM7 Auger line appears as a post-peak after the Co^2^⁺ 2*p*_3/2_ main peak. The Ni 2*p* spectrum (Fig. 3f) confirms the presence of Ni^2^⁺. The main peaks at 854.8 and 872.2 eV correspond to Ni^2+^ 2*p*_3/2_ and Ni^2+^ 2*p*_1/2_, respectively. The peaks with a BE at 859.9 and 803.5 eV are attributed to the satellite peaks. Additionally, the Co LM7 Auger line appears as a pre-peak preceding the Ni^2^⁺ 2*p*_1/2_ main peak, while the Co LM6 Auger line acts as a post-peak following the Ni^2^⁺ 2*p*_1/2_ main peak. Additionally, the Mn LM1 Auger line serves as a post-peak positioned after the Ni^2^⁺ 2*p*_3/2_ main peak. The Mn 2*p* spectrum (Fig. 3g) indicates the presence of Mn^2+^ 2*p*_3/2_ (644.2 eV) and Mn^2+^ 2*p*_1/2_ (655.8 eV). Two Ni LM2 Auger lines appear as a pre-peak and a post-peak, positioned before and after the Mn^2+^ 2*p*_3/2_ main peak, respectively. In Fig. 3h, the spectrum of Zn 2*p* shows the main peaks of Zn^2^⁺ 2*p*_1/2_ and Zn^2^⁺ 2*p*_3/2_ at the BE of 1021.1 and 1044.2 eV. The satellite peak is located at 1040.3 eV.

**Fig. 4:**
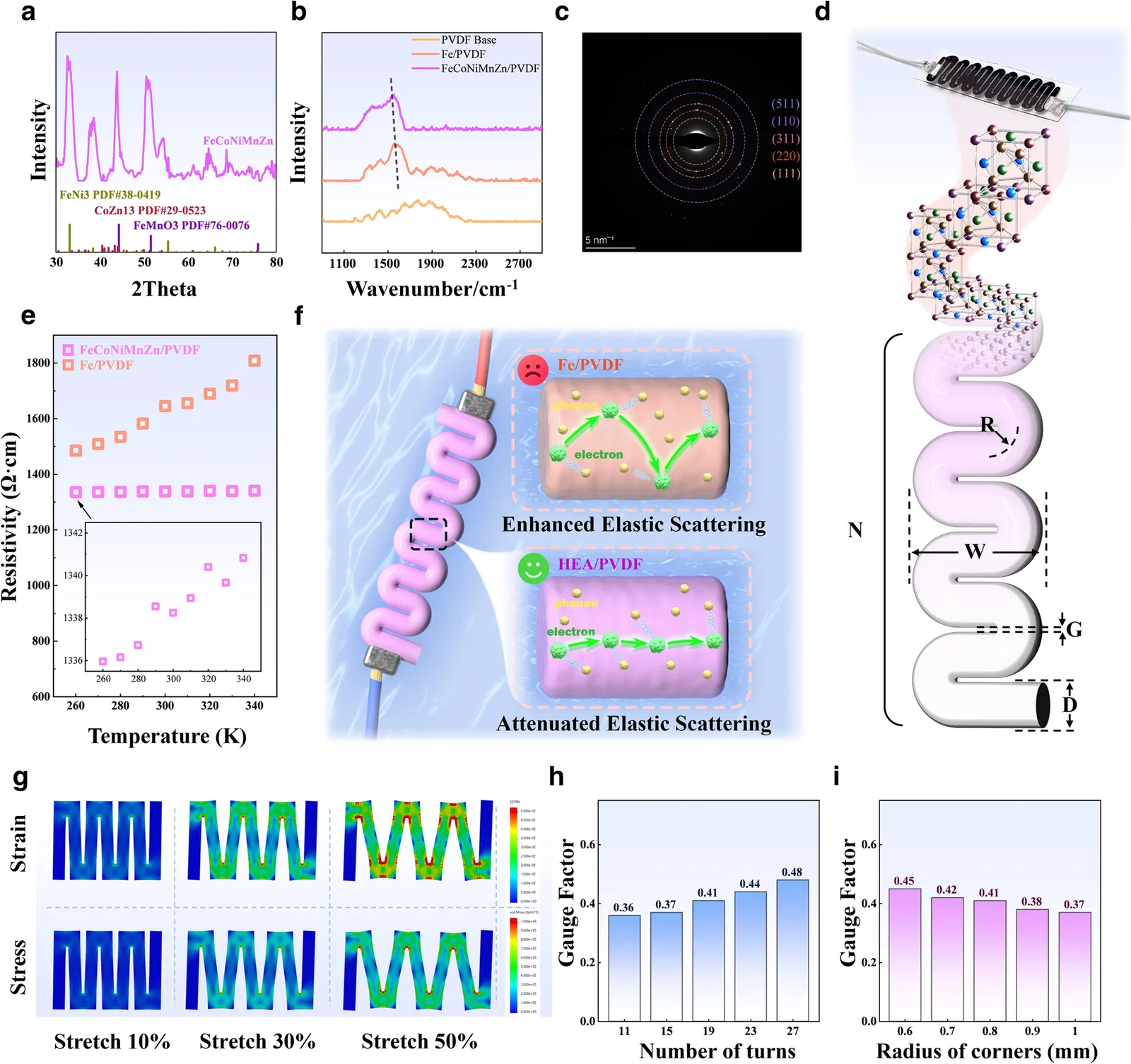
Crystal structure characterization and the low TCR properties of high-entropy alloy fiber. **a** X-ray diffraction (XRD) patterns of high-entropy alloy fibers. **b** Raman spectra of high-entropy alloy fiber, single Mn element fiber, and PVDF fiber substrates. **c** Selected area electron diffraction (SAED) patterns of high-entropy alloy fibers. **d** Structural parameter design of the sensor. **e** Trend of the resistance of FeCoNiMnZn/PVDF fiber and Fe/PVDF fiber in temperature change from 260 to 340 K. **f** Enhanced and attenuated electron–phonon elastic scattering altered electron transport free path during heating. **g** Finite element analysis of the sensor's rotating structure under 10%, 30%, and 50% stretch. **h** Effect of the number of turns on sensor performance. **i** Effect of the corner radius on sensor performance

In typical metallic materials, increased temperatures augment electron–phonon elastic scattering, significantly reducing the mean free path of electrons and resulting in intricate transport channels. This results in a significant elevation in resistivity with an increasing temperature [49, 61, 62]. In comparison, HEAs, consisting of elements with significant variations in atomic radius and chemical characteristics, demonstrate pronounced lattice distortion resulting from the disruption of optimal atomic arrangement [63–65]. The XRD investigation of annealed FeCoNiMnZn/PVDF fibers in Fig. 4a, which exhibits several face-centered cubic (FCC) phases, namely FeNi_3_, CoZn_13_, and FeMnO_3_ (PDF# 38-0419, 29-0523, and 76-0076), indicates diverse lattice distortions. Furthermore, the selected area electron diffraction (SAED) patterns (Fig. 4c) exhibit distinct diffraction rings that correspond to many FCC planes, including (111), (220), (311), (110), and (511). These distortions induce atoms to stray from their optimal lattice locations, diminishing the electron–phonon coupling constant [66, 67]. As a result, the electron–phonon scattering process is diminished, reducing the temperature dependence of resistivity and leading to a lower TCR. Raman spectroscopy results are shown in Fig. 4b. In comparison with single-element samples, the HEA-doped samples exhibited a phonon redshift of roughly 44.9 cm⁻^1^ and a spectrum broadening of around 8.4%, signifying phonon softening and diminished phonon lifespan [68]. The alterations in phonons further diminish elastic scattering, hence reducing its impact on resistivity [62, 69] and contributing to the observed decrease inTCR.

To capitalize on this feature, a rotational‐structured flexible strain sensor using electrohydrodynamic direct writing was designed, with key geometric parameters including the printing diameter D, width W, corner radius R, spacing gap G, and number of turns N. This spiral architecture not only enhances the extensibility of the conductive pathway and its tolerance to deformation, but also effectively modulates stress distribution and sensing performance (Fig. 4d). As shown in Eqs. 1 and 2, the total fiber length L and sensor length L’ can be calculated. Moreover, smaller printing diameter D and spacing gap G improve the spatial resolution of the sensing region and boost strain sensitivity, while width W and the number of turns N together determine the total conductive‐path length and allowable deformation range; variations in corner radius R markedly influence strain‐transfer efficiency and structural stability. It is worth emphasizing that this design is not merely intended to optimize mechanical response but is fundamentally aimed at achieving a low TCR, enabled by the incorporation of additional high‐entropy alloy fibers and the uniform distribution of the conductive pathway.

1 $$L = \sum\limits_{n = 1}^{N} {\left[ {2R(\pi - 2) + 2W} \right]} + W$$

2 $$L\prime = \mathop \sum \limits_{n = 1}^{N} \left[ {2\left( {D + G} \right)} \right] + D$$

3 $$\alpha = \frac{1}{{R_{0} }}\left. {\frac{{{\mathrm{d}}R}}{{{\mathrm{d}}T}}} \right|_{{T = T_{0} }} \approx \frac{{R\left( T \right) - R\left( {T_{0} } \right)}}{{R_{0} \left( {T - T_{0} } \right)}}$$

To elucidate the correlation between the deformation mechanism and resistance variation, the strain and stress distributions of the sensor under overall strains of 10%, 30%, and 50% were simulated by the finite element method, as shown in Fig. 4g. When the overall structure is stretched, stress and strain are highly concentrated at the corner regions of the rotational-structured elements, while the straight segments experience relatively low strain. This non-uniform strain distribution is central to the deformation mechanism of the serpentine geometry. Such a specific strain pattern directly determines the resistance variation of the sensor.

According to the simulation results, the sensitive material located at the corner regions, which undergoes tensile strains far exceeding the average, initiated microcrack formation and propagation first. This led to the premature rupture of conductive pathways in these regions and a significant increase in resistance. The local microcracks induced by structural deformation were the dominant factors causing resistance changes [70–72]. In contrast, the straight segments, experiencing lower strain, largely retained their conductivity. Therefore, the overall resistance change of the sensor is primarily attributed to contributions from these high-strain concentrated regions. According to the indications from finite element analysis, the corner radius R and the number of turns N in the rotational structure of the strain sensor were adjusted, and comparative experiments were conducted under 30% strain. As shown in Fig. 4h, the gauge factor (GF) of the sensor increased with the number of turns. In contrast, enlarging the turning radius exerted an opposite effect on the GF (Fig. 4i). Considering their combined influence on the overall sensor length, designers can select appropriate turn numbers and turning radius to achieve tunable control over the sensing performance.

The resistance measurements across the temperature range from 260 to 360 K are illustrated in Fig. 4e. The TCR *α*, calculated in Eq. 3, revealed a significantly lower value of 45.59 ppm K^−1^ for the HEA sample, in contrast to 2207.38 ppm K^−1^ for the single-element variant. To further investigate the peak shift induced by lattice distortion, XRD patterns, thermogravimetric analysis, and thermomechanical analysis were conducted for single-element Fe/PVDF, binary FeNi/PVDF, and HEA/PVDF samples, as shown in Figs. S9–S11. The TCR of the sample was measured and is compared in Fig. S12. The results validated that lattice deformation and phonon softening significantly inhibit electron–phonon scattering. Consequently, the electron mean free path is less influenced by thermal heating, in contrast to common metals. The abbreviated yet stable electron transport channels contributed to resistivity stability during heat fluctuations (Fig. 4f), facilitating the remarkably low TCR.

### Performance Characterization of Strain Sensor

**Fig. 5:**
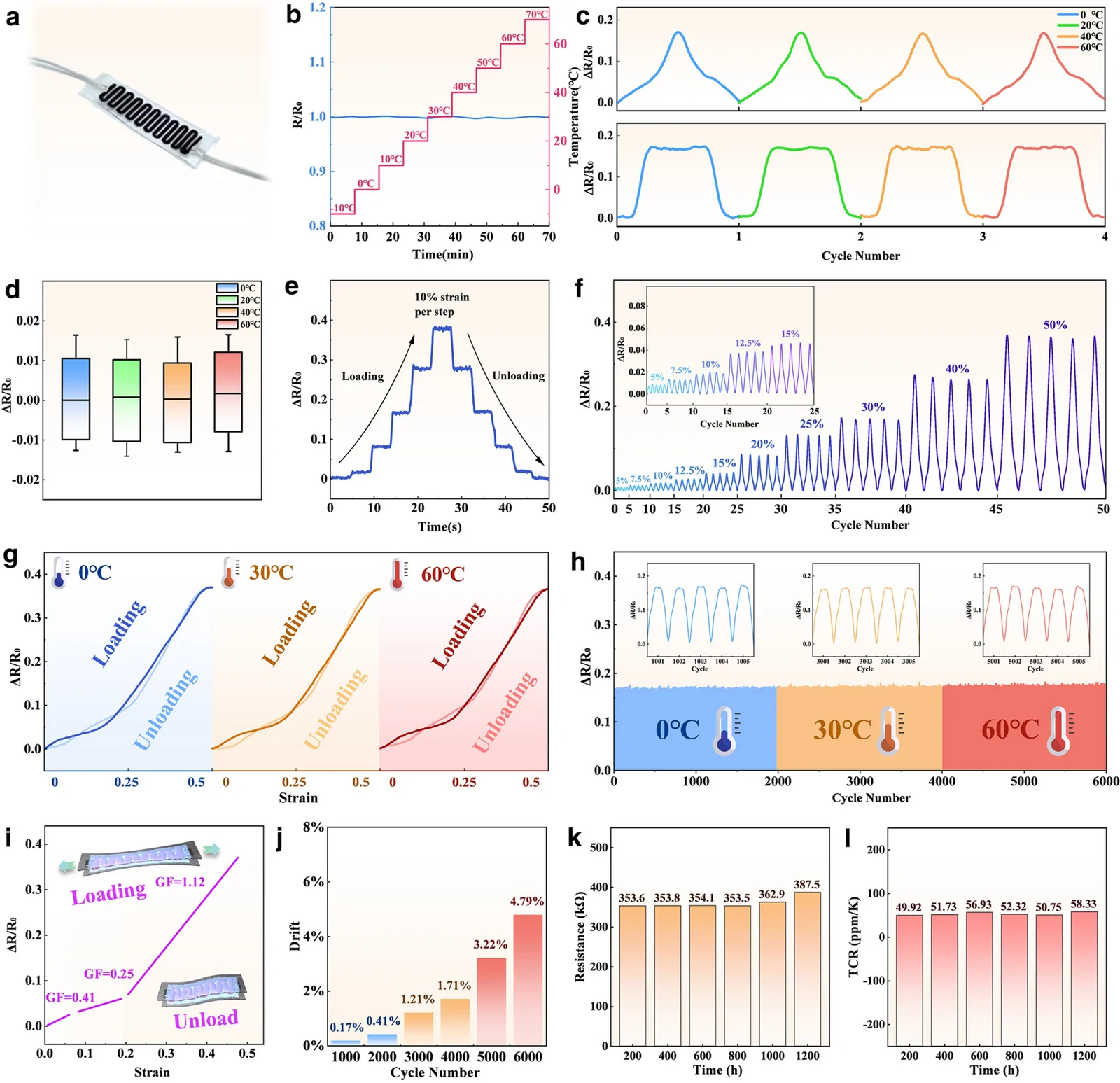
Testing the tensile properties of strain sensors. **a** Schematic diagram of the strain sensor. **b** Resistance stability: the temperature rises from -10 to 70 °C with a gradient of 10 °C in 70 min. **c** 30% Tensile strain curves and response time curves at 0℃, 20℃, 40℃ and 60℃. **d** Temperature stability of the standing sensor at 0 °C, 20 °C, 40 °C, and 60 °C. **e** Continuous loading and unloading with a gradient of 10% from 10 to 50% strain. **f** Resistance change of strain sensor during loading and unloading at 5 ~ 50% strain. **g** Resistance change of loading and unloading at strain of 50% at 0 °C, 30 °C, and 60 °C. **h** Strain sensor within 6000 cycles at a strain of 30% at 0 °C, 30 °C, and 60 °C. The insets show the relative change in resistance for specific cycles during the cycles. **i** The strain sensor with GF of 0.41 for the strain range from 0% to 6.5%, GF of 0.25 for the strain range from 6.5 to 19.2%, and GF of 1.12 for the strain range from 19.2 to 50%. **j** Drift measurement during the 6000 cycles' stability testing. **k** Degradation of the sensor's intrinsic resistance value after 1200 h. **l** Degradation of the sensor's TCR value after 1200 h

The fabricated sensor was tested under varying temperature conditions using a universal tensile testing machine. The sensor was stretched at a specified rate and displacement, and the tensile performance is presented in Fig. 5.

The schematic of the sensor subjected to compression testing is illustrated in Fig. 5a, where the change of resistance is measured by applying different strains to the sensor inside the fixture at varying and constant room temperatures. To evaluate the thermal stability and TCR of the sensor, a stationary sensor was tested under temperature variations ranging from − 10 to 70 °C in 10 °C increments in Fig. 5b, and no obvious baseline shift and significant changes in resistance were observed throughout the process. The surface temperature distribution of the sensor placed on the heating stage under different set temperatures was obtained using an infrared thermal imager. At the same time, the corresponding real-time resistance values were synchronously recorded using an LCR digital bridge. These results are provided in Fig. S13 and Table S3. In dynamic stretching, 30% strain tests were performed at 0, 20, 40, and 60 °C. The resistivity changing rates at different temperatures exhibited a high degree of consistency in the stretch-return curves and the response test curves (Fig. 5c), which had imperceptible deviations in magnitude and a stable 310 ms rising-edge response time and 350 ms falling-edge response. Static measurements at 0, 20, 40, and 60 °C in Fig. 5d revealed that the sensor maintains nearly constant resistivity, underscoring its outstanding temperature immunity. The fabricated strain sensor exhibited excellent electrical and mechanical properties at room temperature. The gradient stability at each strain stage is verified in Fig. 5e. The sensitivity and resolution in the low (5%–15%) and medium (20%–50%) strain ranges during loading and unloading are shown in Fig. 5f. In Fig. 5g, during the 50% strain loading and unloading processes at 0, 30, and 60 °C, the sensor exhibited consistent resistance variation trends and amplitudes. This result further confirmed the sensor’s thermal stability and intrinsic temperature immunity, which is a critical advantage for applications requiring stable performance in environments with temperature fluctuations. The gauge factor is defined in Fig. 5i, which GF = 0.41 at strain range from 0 to 6.5%, GF = 0.25 at strain range from 6.5% to 19.2% and GF = 1.12 at strain range from 19.2% to 50%. The diagrams illustrating the mechanical properties of strain-tension and latent strain–stress are presented in Figs. S14 and S15. The verification of cyclic stability is presented in Fig. 5h, and the representative cyclic periods are shown in Fig. S16. After 6000 cycles of 30% continuous strain at 0, 30, and 60 °C, no significant failure or deviation was observed, demonstrating the sensor's long-term stability and reliability.

To further investigate performance degradation during fatigue testing, a full 6000-cycle experiment was conducted. Using the mean strain extrema of the first 100 tensile cycles as the baseline (Fig. 5h) and extracting quantified resistance drift data every 1000 cycles. In Fig. 5j, the quantified resistance drift increases with the number of cycles, confirming the progressive degradation of performance. It is noteworthy that the degradation rate was not constant. Specifically, the resistance drift during the last 2000 cycles (1.54% per thousand cycles) was significantly higher than that observed in the first 4000 cycles (0.43% per thousand cycles), suggesting that the material exhibits a certain degree of damage tolerance and stability in the later stages of cycling. These results supported the conclusion in the main text that the material demonstrates excellent durability under complex operating conditions and provided critical evidence for quantifying its long-term service lifetime. Additionally, the durability tests exceeding 1200 h are shown in Fig. 5k, l. The experimental data indicated that the intrinsic resistance of the sensor remained highly stable throughout the entire testing period (Fig. 5k), with only a slight maximum increase of 9.6% observed at 1200 h. In Fig. 5l, the TCR exhibited only a minor variation of up to 12.74 ppm K^−1^ (compared with the initial value of 45.59 ppm K^−1^), which still represented a relatively low TCR, demonstrating good thermal stability. The minimal changes in these two key parameters clearly confirm that even after 1200 h of continuous testing, the HEA nanofibers did not show any significant degradation in electrical performance.

A quantitative comparison with existing low-TCR flexible strain sensors is provided in Table 1. The comparison of the applicable temperature ranges for other sensors with low resistance coefficient characteristics is presented in Fig. S17. The performance of silver nanowires, graphene composites, carbon nanotubes and metallic systems was summarized and presented with respect to key parameters such as TCR, strain range, GF, and stability.

**Table 1:** Comparison with low TCR strain sensors in the latest research

Materials of Sensitive Layer	TCR (ppm K^−1^)	Strain range (%)	GF	Temperature range (°C)	Stability (cycles)	References
AgNWs, SWCNTs	221	80	3.39	− 20–130	20000	[73]
Gold, SWCNTs, FLG	160	80	2415.76	0–60	2000	[74]
CNT, GNP, GCE	114	100	14550.2	0–100	10000	[28]
A-POSS, GO-CNT	16.67	0.04	25000	− 20–100	100	[75]
Pb_2_Ru_2_O_6_, TiB_2_	281	± 0.03	19.8	100–700	–	[76]
AgNPs, SiO_2_	30	0.3	16.4	10–60	3600	[35]
Mo, W, Nb Alloy	> 500	0.45	448.5	− 150–1100	1200	[77]
This work	45.59	50	1.12	− 10–70	6000	–

Based on these comparative data, the detection of large strains (> 50%), the sensor developed in this study, employing a high-entropy alloy as the sensing layer, maintains a relatively low TCR value. In contrast, sensors designed for small-strain detection (< 1%) could achieve a wider applicable temperature range and higher sensitivity, but their strain detection range is extremely limited. This systematic comparison strongly highlighted the novelty and advancement of our work, demonstrating an outstanding balance between wide strain range and low temperature sensitivity, and providing an effective solution to the challenge of simultaneously addressing temperature fluctuation interference and broad deformation monitoring in practical applications.

### Application of Wearable System

**Fig. 6:**
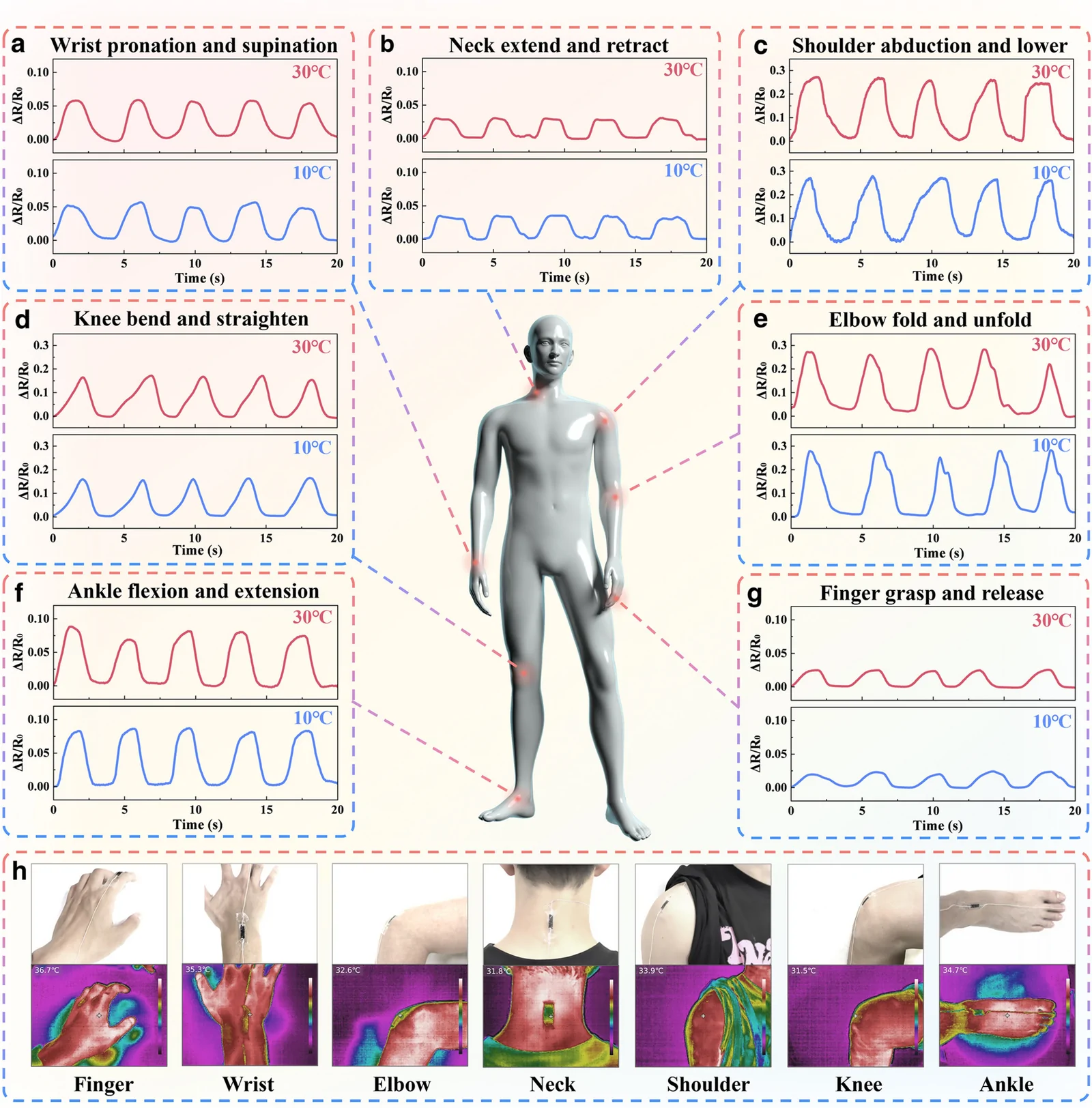
Application of strain sensors in wearable devices. The collection of strain signals by sensors mounted on the **a** wrist, **b** neck, **c** shoulder, **d** knee, **e** elbow, **f** ankle, and **g** finger joints of the human body. **h** Optical and infrared thermal images of the sensor worn on human joints

A flexible strain sensor was developed to demonstrate excellent wearability and stable performance across varying temperature conditions. The sensor is lightweight, soft, and highly conformable, adhering closely to human skin and ensuring stable operation even at highly dynamic regions such as joints. The low power consumption and high sensitivity enable long-term monitoring, maintaining signal stability in daily environments with fluctuating temperatures. To evaluate its practical applicability, sensors were installed on several human joints, including the wrist, neck, shoulder, knee, elbow, ankle, and finger. The installation method is shown in Fig. S18. Strain detection experiments were conducted at ambient temperatures of 10 and 30 °C. As shown in Fig. 6a–g, the resistance of the sensor increased with joint flexion due to the mechanical strain. The magnitude of resistance change reflected the sensor’s sensitivity to deformation. Optical and infrared images of the wearable device mounted on human joints are provided in Fig. 6h, further supporting the sensor’s capability in practical scenarios.

The experimental results, together with the preceding performance tests, confirmed that the sensor possessed sufficient resolution to distinguish different joint motion angles, with clear differentiation observed both across varying amplitudes of different joints and among distinct motion angles of the same joint. Under varying temperature conditions, sensors mounted on each body part individually exhibited consistent signal trends and comparable amplitude responses, without baseline drift, thereby demonstrating high thermal stability and reliable signal performance. In Fig. S19, by collecting and analyzing the output signals, the system enabled stable and accurate detection of joint motion in complex thermal environments. This capability supported real-time assessment of movement and physical condition in practical wearable applications.

### Wide-Temperature-Range Manipulator Strain Detection System

**Fig. 7:**
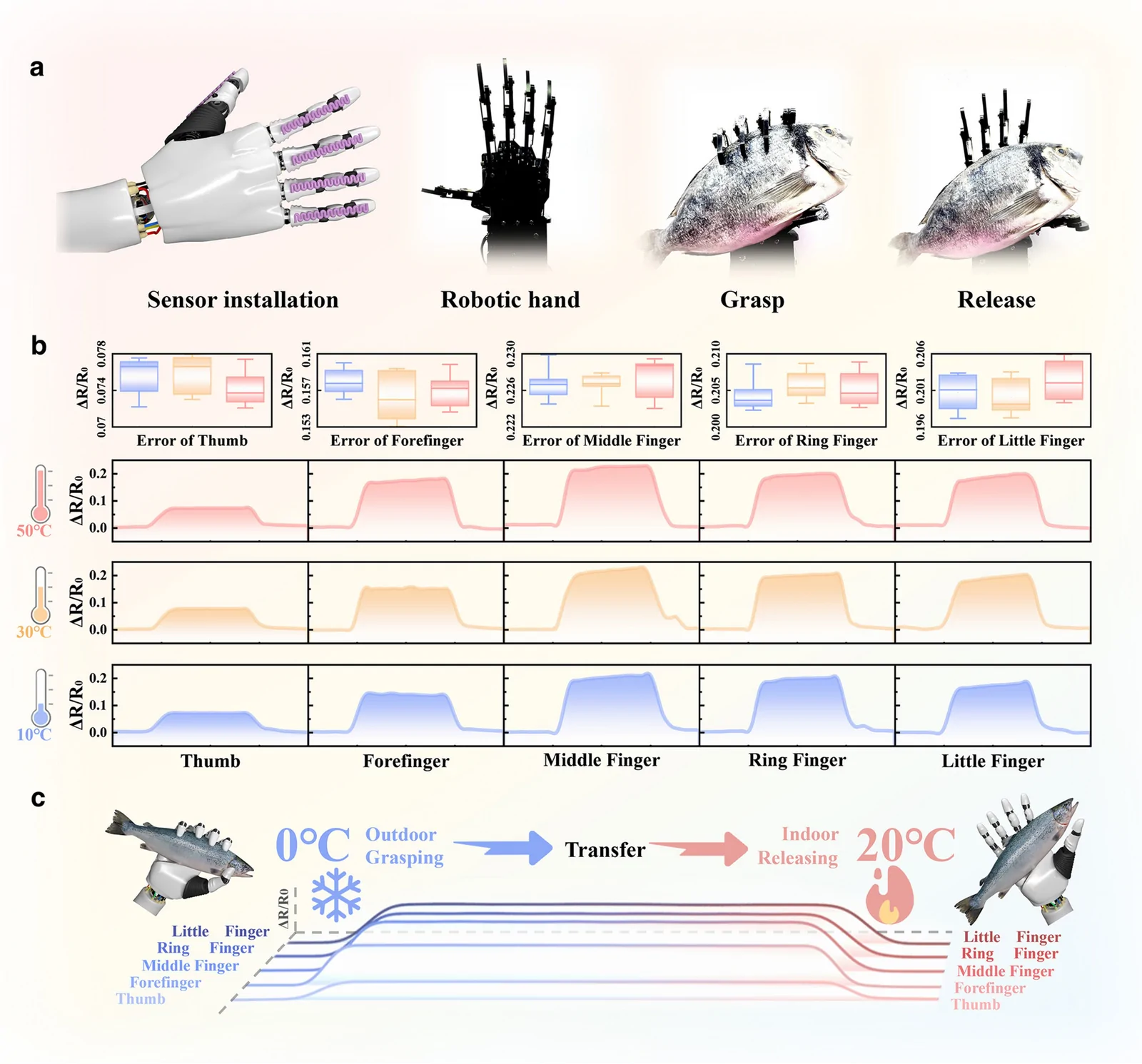
Strain detection system for robotic hand. **a** Strain sensor mounted on a robotic hand, and gripping schematic. **b** Comparison of signals and errors of grasping at different temperatures. **c** Strain detection of grasping action under varying environmental temperatures

The designed flexible strain sensor was attached to the finger joints of the robotic hand, as depicted in Fig. 7a, to assess its strain stability for detecting grasping across varying environmental temperatures. The experiment was performed at environmental temperatures of 10, 30, and 50 °C, with the robotic finger grabbing the same object ten times to replicate real operational settings (Fig. 7b). The box plots indicate that the sensors positioned at various locations of the robotic finger joints exhibit maximum severe errors of 0.59%, 0.7%, 0.59%, 0.66%, and 0.88%, with maximum average value errors of 0.16%, 0.18%, 0.09%, 0.16%, and 0.21%, respectively. In addition, a dynamic temperature variation experiment is conducted in Fig. 7c. The robotic arm is equipped with fingertip-mounted sensors, grasped an object in a low-temperature environment of 0 °C, and subsequently transferred it to a room-temperature environment of 20 °C for release. Throughout this transition, the strain signals remained clear and stable, without any noticeable baseline drift or signal distortion caused by the environmental temperature change. The resistance peaks corresponding to the grasping and releasing events were accurately preserved. This dynamic thermal scenario verified the sensor’s outstanding environmental adaptability under realistic and complex operating conditions. The results demonstrate that the engineered flexible strain sensors possessed exceptional signal stability and reproducibility across varying temperature environments, rendering them appropriate for strain monitoring of robotic fingers in intricate thermal situations. This offers dependable technical assistance for attaining high-precision, low-power flexible electronic systems.

## Conclusions

This study presents a temperature-immune flexible strain sensor fabricated by electrohydrodynamic direct writing of FeCoNiMnZn high-entropy alloy fibers and subsequent annealing-induced metallization within the PVDF fiber matrix. The sensor exhibited a gauge factor of 1.12 at 50% strain, a rapid response time of 310 ms, and excellent cycling stability over 6000 loading–unloading operations without baseline drift. Owing to the intrinsic lattice distortion and phonon softening effects of the high-entropy alloy, the device achieved a low TCR of 45.59 ppm K^−1^ across − 10 to 70 °C, ensuring signal stability under fluctuating thermal environments. The temperature-immune characteristics and flexibility of the device enabled reliable strain monitoring in multiple scenarios, including human joint motion detection, wearable health monitoring, and robotic manipulation, underscoring its adaptability in complex conditions. The strategy of employing high-entropy alloys as thermally stable conductive frameworks provides a viable route to overcome the limitations of conventional composite compensation methods. Moreover, as a direct-writing technique, this approach is highly compatible with mature industrial processes such as inkjet printing and roll-to-roll manufacturing, allowing seamless integration into existing flexible electronics production lines for patterned circuit fabrication without complex transfer steps. Overall, this study not only advances the development of intrinsically stable flexible sensors but also lays a solid foundation for next-generation wearable electronics and flexible sensor systems capable of long-term operation in thermally dynamic environments.

## Supplementary Information

Below is the link to the electronic supplementary material.

Supplementary file 1 (DOCX 8237 KB)

Supplementary file 2 (MP4 4711 kb)
