# Supplementary material for: Temperature-Immune High-Entropy Alloy Flexible Strain Sensor on Electrospinning Nanofibrous Membrane
Source: Nanomicro Lett. 2026 Jan 12;18:191. doi: 10.1007/s40820-025-02033-3 (PMC12791098; doi:10.1007/s40820-025-02033-3)
Supplement: Supplementary file 1 — (DOCX 8237 KB) [file 40820_2025_2033_MOESM1_ESM.docx]

Supporting Information for

**Temperature Immune High Entropy Alloy Flexible Strain Sensor on Electrospinning Nanofibrous Membrane**

Wenxin Li^2, #^, Xianruo Du^1, #^, Yisheng Zhong^3^, Ruixin Chen^2^, Yuyang Wang^2^, Huatan Chen^4^, Huangping Yan^2^, Yifang Liu^2^, Chentao Zhang^2,^ * and Gaofeng Zheng^2,^ *

^1^ College of Physical Science and Technology, Xiamen University, Xiamen 361005, P. R. China

^2^ Pen-Tung Sah Institute of Micro-Nano Science and Technology, Xiamen University, Xiamen 361100, P. R. China

^3^ State Key Laboratory of Flexible Electronics Technology, Tsinghua University, Beijing, 100084, P. R. China

^4^ School of Mechanical and Automotive Engineering, Xiamen University of Technology, Xiamen 361024, P. R. China

^#^Wenxin Li and Xianruo Du contributed equally to this work.

*Corresponding authors. E-mail: [zhangct@xmu.edu.cn](mailto:zhangct@xmu.edu.cn) (Chentao Zhang); [zheng_gf@xmu.edu.cn](mailto:zheng_gf@xmu.edu.cn) (Gaofeng Zheng)

S1 Supplementary Figures


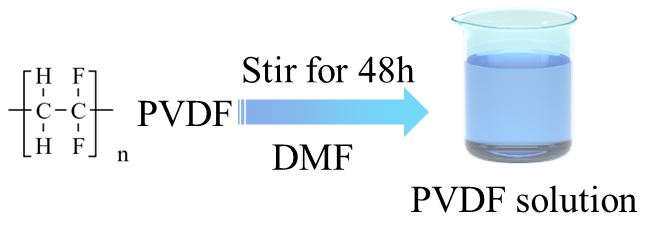


**Fig. S1** Schematic diagram of PVDF solution preparation

In this step, 1 gram of PVDF with a molecular weight of 600,000 was dissolved in 6.7 grams of DMF solution with a purity of 99.9%, and was stirred on a magnetic stirrer for 48 hours to ensure complete dissolution.


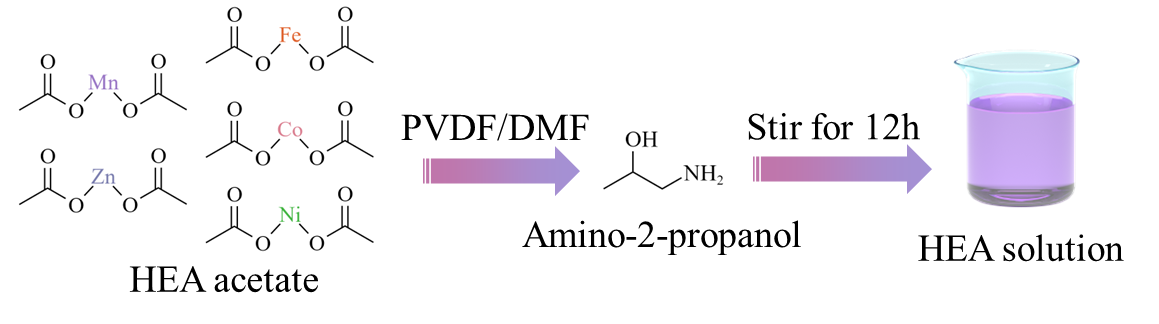


**Fig. S2** Schematic diagram of HEA solution preparation

Different acetate salts of metal elements with different molar masses and 1.8g of isopropylamine were added to the uniformly dissolved PVDF/DMF solution (with a mass ratio of 1g:8.2g). The mixture was stirred at room temperature for 24 hours to ensure the formation of a uniform high-entropy alloy precursor solution.

**
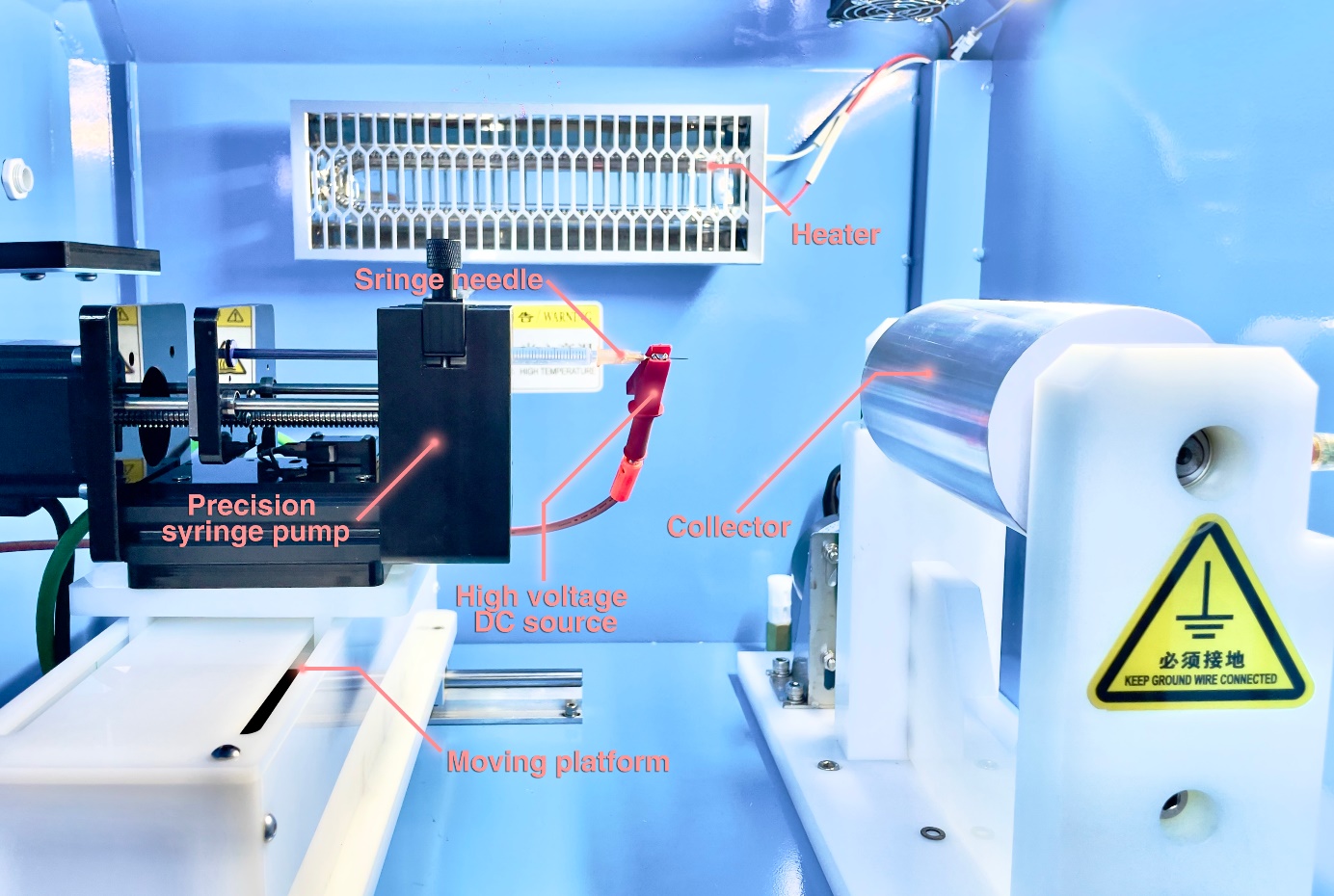
**

**Fig. S3** The electrospinning platform for preparing the substrate


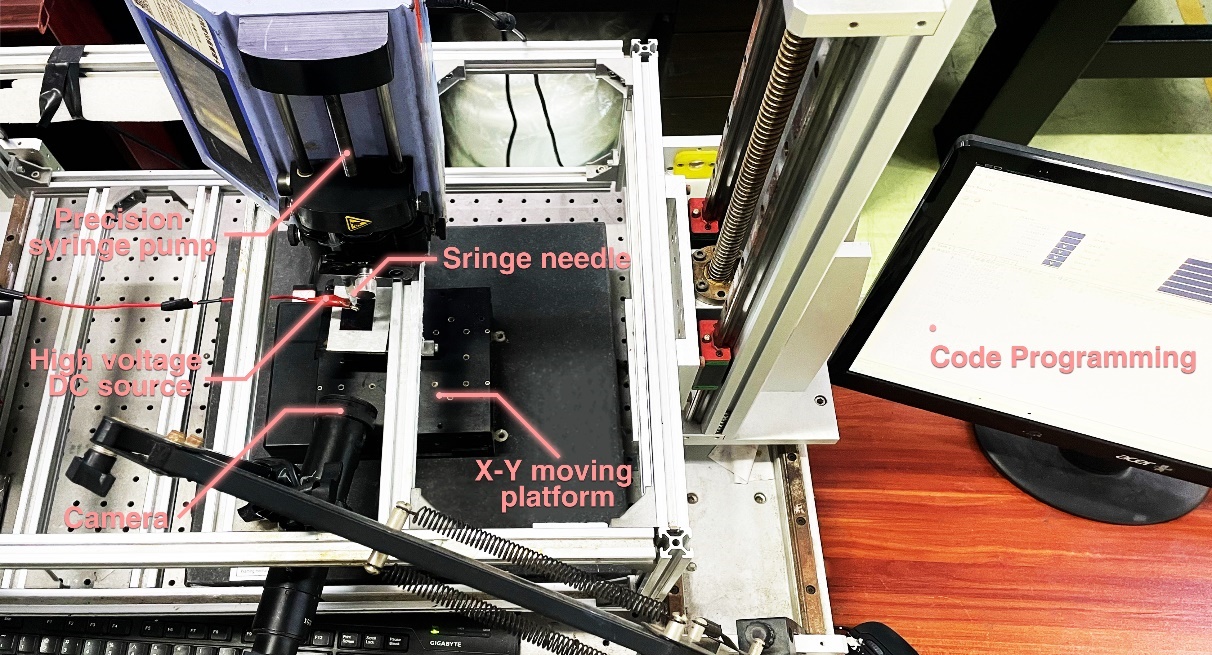


**Fig. S4** The electrohydrodynamic direct writing platform for sensor fabricating

Through the coordinated integration of a precision syringe pump, an XY motion platform, a high-voltage power supply, and a monitoring camera, electrohydrodynamic direct writing enables the accurate deposition of predesigned fiber patterns onto the substrate. This synergy allows precise fiber placement and reproducible fabrication of complex patterns.


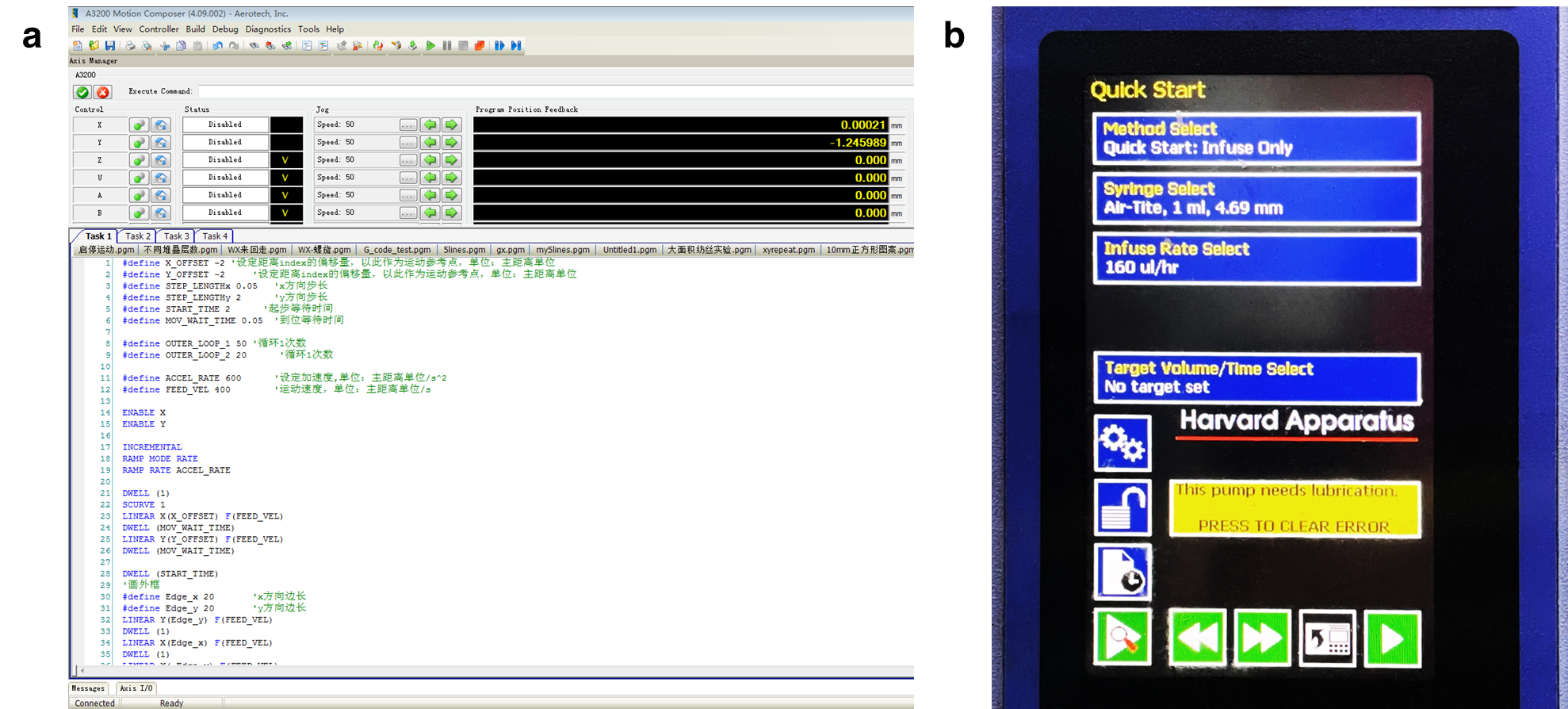


**Fig. S5** The image of software used in fabrication. a The Motion platform software and b the software of precision syringe pump

Precise control of the motion platform through programming, together with regulated syringe pump flow, enables accurate regulation of the electrohydrodynamic direct writing process for stable fiber deposition.


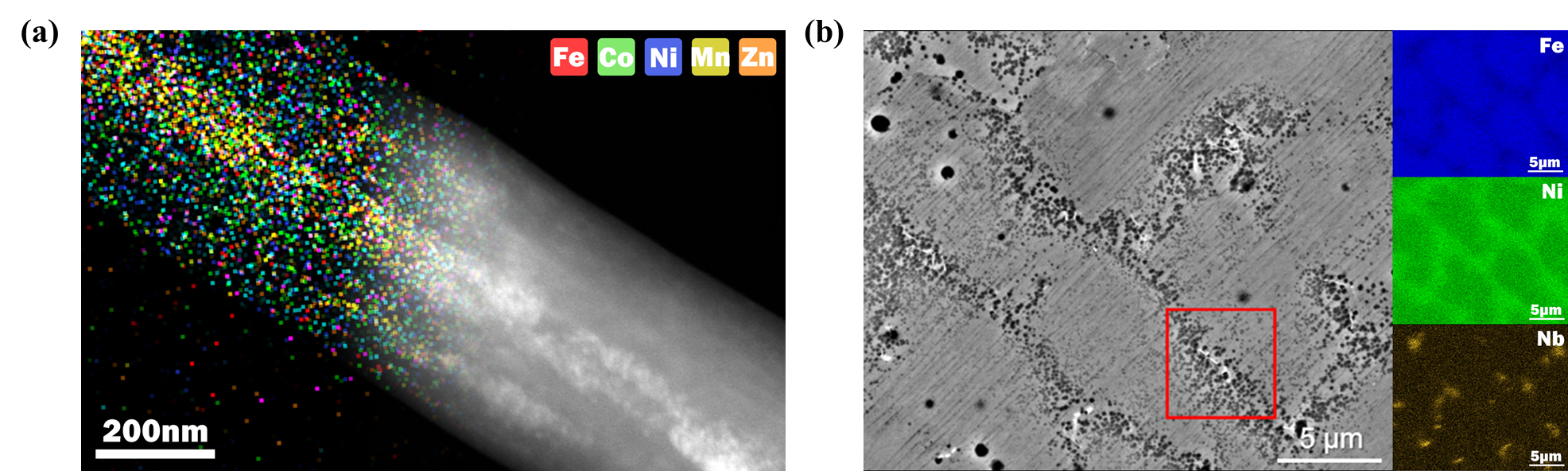


**Fig. S6** The atomic diffusion efficiency test by EDS line scans. **a** The microstructure and EDS line scan results of the nanofibers prepared in this paper. and **b** the alloy prepared by laser sintering of particles [S1].

In Fig. S6a, the compositional distribution was analyzed using EDS elemental mapping at a 200 nm scale. The results confirm that, after the electric-field-induced polarization treatment, the five metallic elements are uniformly distributed, with no evidence of compositional segregation or elemental agglomeration.

To further substantiate, the results were compared with the alloy prepared by the laser particle sintering method reported by Zhang et al., as shown in Fig. S6b. In that work, TEM and EDS analyses clearly revealed compositional fluctuation particles at the 5 μm scale along with well-defined grain boundaries, indicating pronounced segregation and agglomeration phenomena. In contrast, the material obtained in this study maintains compositional uniformity at the sub-nanometer scale without visible grain boundaries. This direct comparison highlights the significant advantage of our approach in suppressing compositional segregation, thereby strongly supporting the related conclusions presented in the manuscript.


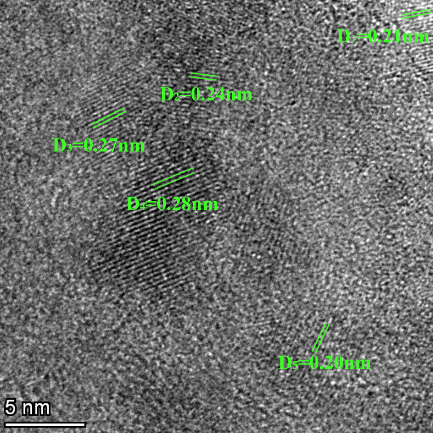


**Fig. S7** STEM interplanar spacing of high-entropy alloy fibers


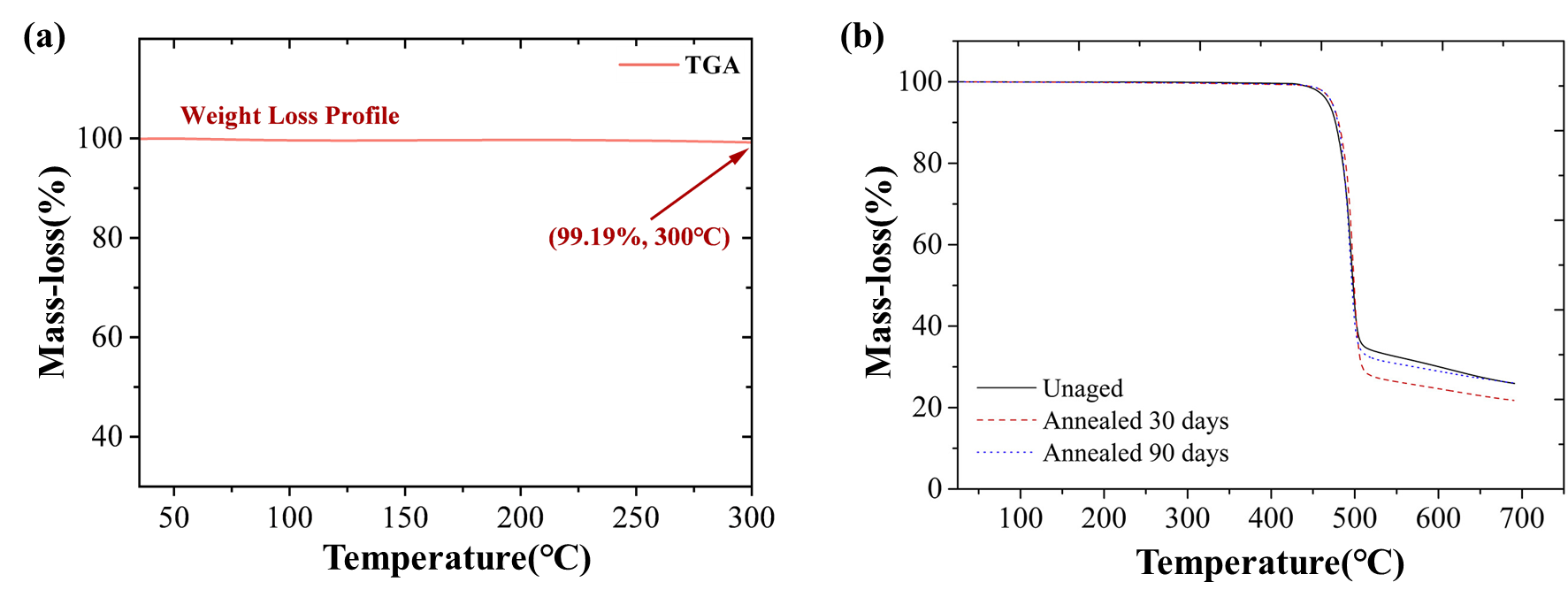


**Fig. S8** Thermogravimetric analysis of PVDF materials. **a** Thermogravimetric analysis curve of electrospun PVDF fiber membrane. **b** Thermogravimetric analysis curve of melt-prepared PVDF panel [S2]

Since the HEA precursors require an annealing process to form a stable alloy lattice structure, conventional polymers such as PU, PEO, or PVA tend to undergo thermal degradation or structural collapse under high temperatures, making it difficult to maintain continuous fibrous morphology.

In contrast, thermogravimetric analysis TGA results show that PVDF membrane exhibits negligible weight loss up to 300 °C under an inert atmosphere, indicating outstanding thermal stability (Fig. S8a). As further demonstrated in the study by KK (Fig. S8b), PVDF does not undergo pyrolysis or show significant mass loss below 400 °C [2]. This property enables PVDF to retain the integrity of its molecular chains during metallization annealing, thereby allowing the formation of the HEA lattice while preserving the flexibility and structural continuity of the polymer matrix.


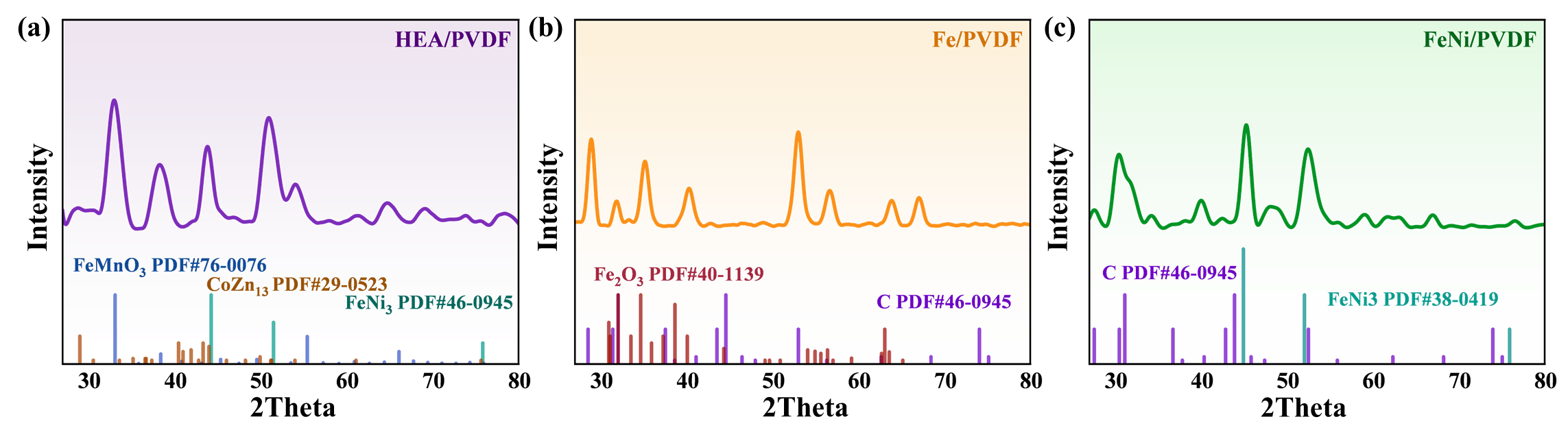


**Fig. S9** XRD analysis of electrospun samples of single metal, binary alloy, and high-entropy alloy. **a** XRD pattern of the HEA/PVDF sample. **b** XRD pattern of the Fe/PVDF sample. **c** XRD pattern of the FeNi/PVDF sample

As shown in Fig. S9, compared with the typical single-phase structures exhibited by the single-metal (Fig. S9b) and binary-metal samples (Fig. S9c), the HEA sample (Fig. S9a) displays broadened and multiple diffraction peaks. Such peak broadening is a characteristic manifestation of the high-entropy effect, arising from lattice distortion induced by the atomic size differences among multiple principal elements. This phenomenon lays the foundation for the superior mechanical properties of the HEA material.


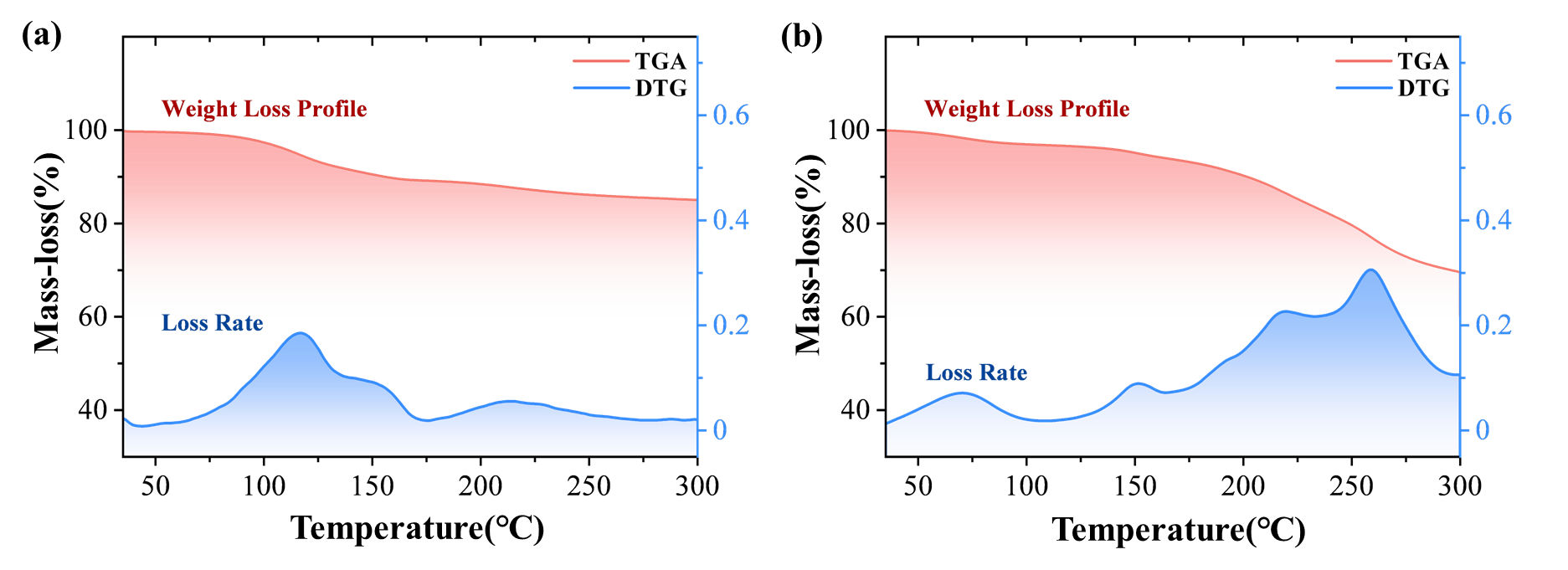


**Fig. S10** TGA tests curves. **a** TGA tests of the Fe/PVDF sample and **b** HEA/PVDF sample

From the TGA analysis of the Fe/PVDF sample in Fig. S10a, it can be observed that weight loss occurs earlier than in the HEA/PVDF sample shown in Fig. S10b, which is attributed to the intrinsic differences in lattice formation energy between the two filler materials. During the sintering process of material preparation, the energy barrier required to form the crystalline structure of single-metal Fe is relatively low. In contrast, the high-entropy alloy consists of five elements, and its lattice formation involves the cooperative diffusion and ordering of multiple atoms under severe lattice distortion. This process is characterized by higher mixing entropy and greater activation energy for lattice formation.


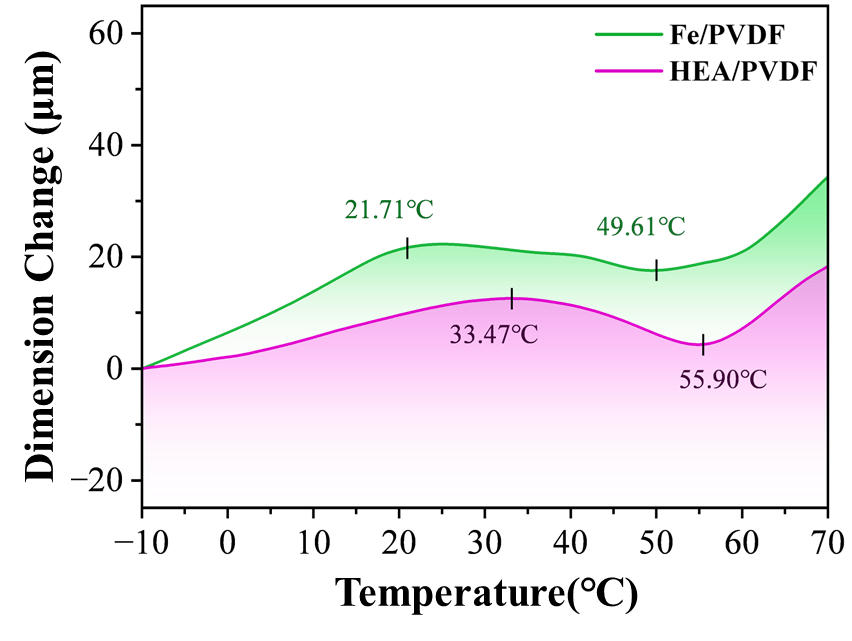


**Fig. S11** TMA tests of the Fe/PVDF sample and HEA/PVDF sample

Thermomechanical analysis (TMA) was conducted on Fe/PVDF and HEA/PVDF samples with a length of 7 mm to investigate their deformation under different temperature conditions. As shown in Fig. S11, significantly smaller deformation was exhibited by the HEA/PVDF sample during the heating process compared with the Fe/PVDF sample. This was further demonstrated as evidence of its thermal dimensional stability, which is regarded as a critical advantage for sensor applications requiring stable performance in environments with temperature fluctuations.


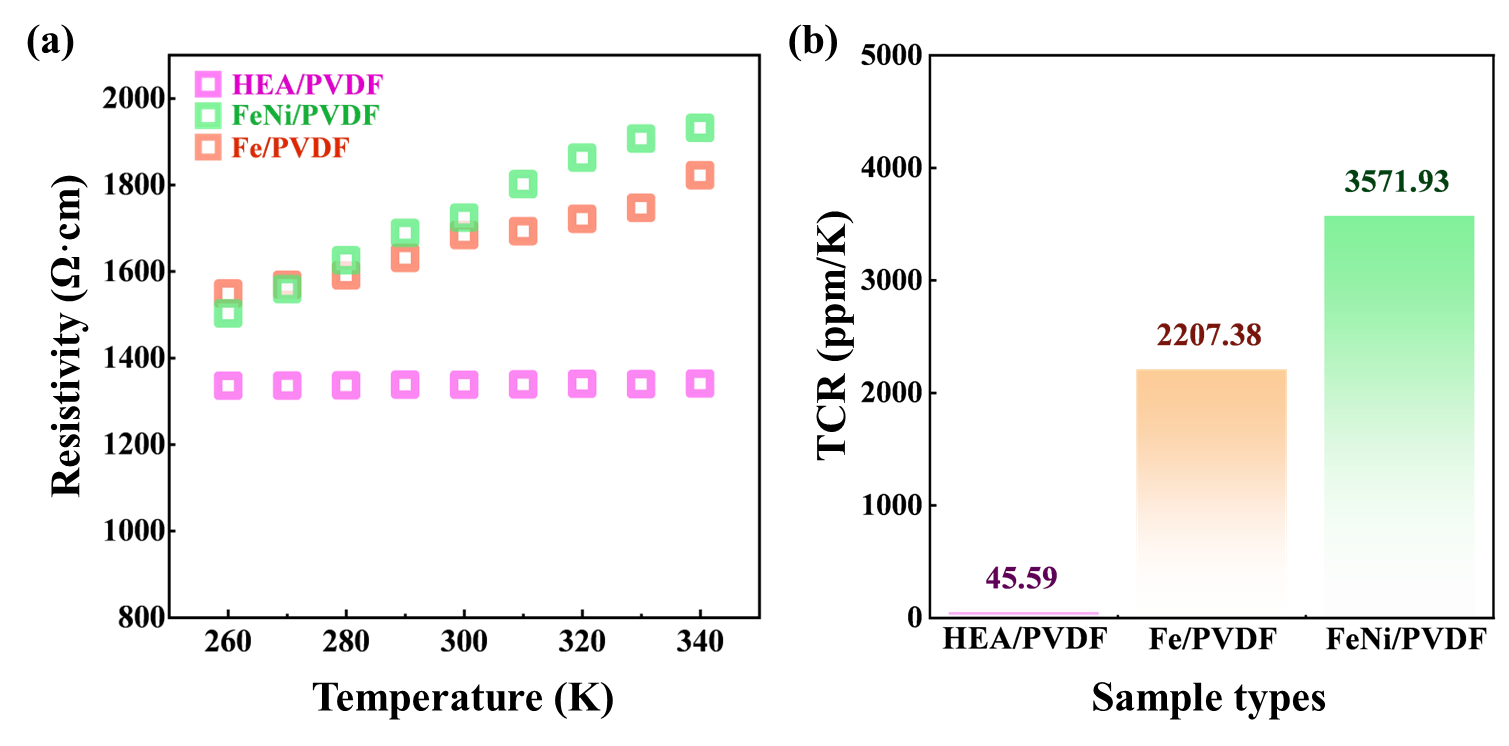


**Fig. S12** Thermal stability of electrical conductivity in single-metal, binary-alloy, and high-entropy-alloy samples. **a** Electrical conductivity at different temperatures. **b** Comparison of the TCR values

By measuring the temperature coefficient of resistance of the samples, the differences are presented in Fig. S12. Benefiting from the unique multi-principal solid-solution structure of the high-entropy alloy, the pronounced lattice distortion and complex electronic interactions effectively regulate its band structure and electron transport behavior, thereby achieving thermoelectric stability far superior to that of simple metals or conventional alloys.


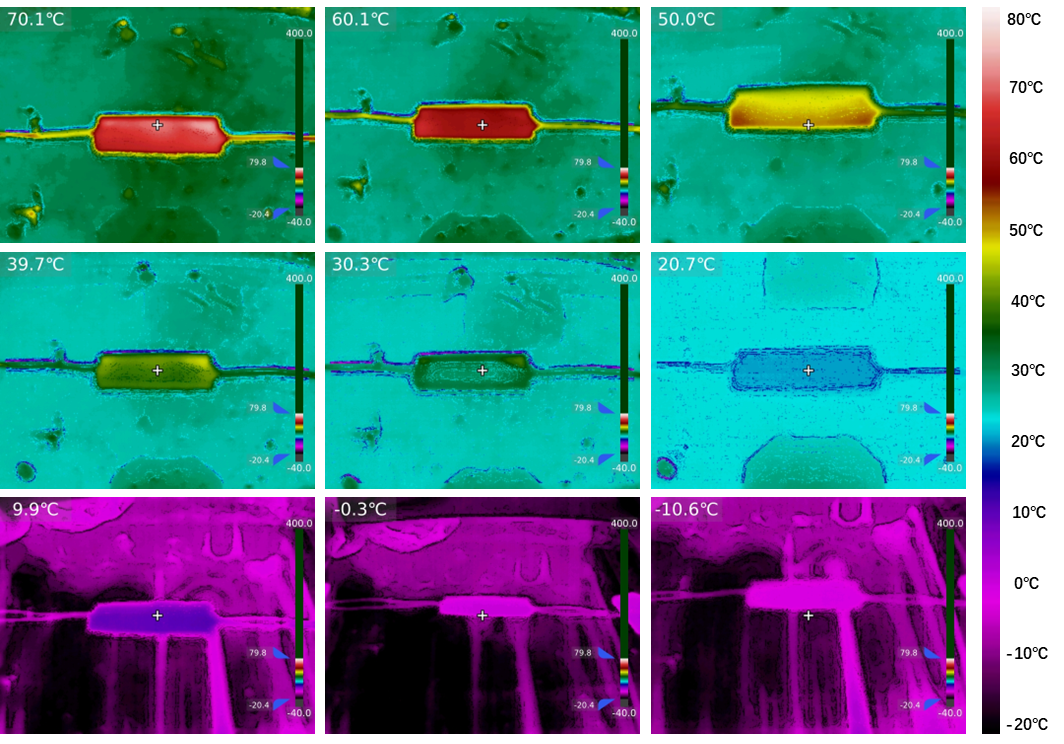


**Fig. S13** Infrared thermal images of the sensor placed on a variable-temperature stage ranging from –10 °C to 70 °C

As shown in Fig. S13, the infrared thermal images clearly reveal the heating condition of the sensor within the specified temperature range. It is worth noting that, due to the specular reflection caused by the smooth surface of the variable-temperature stage, the infrared camera may not accurately capture the surface temperature of the stage. However, this issue does not affect the sensor placed on top of it.

**
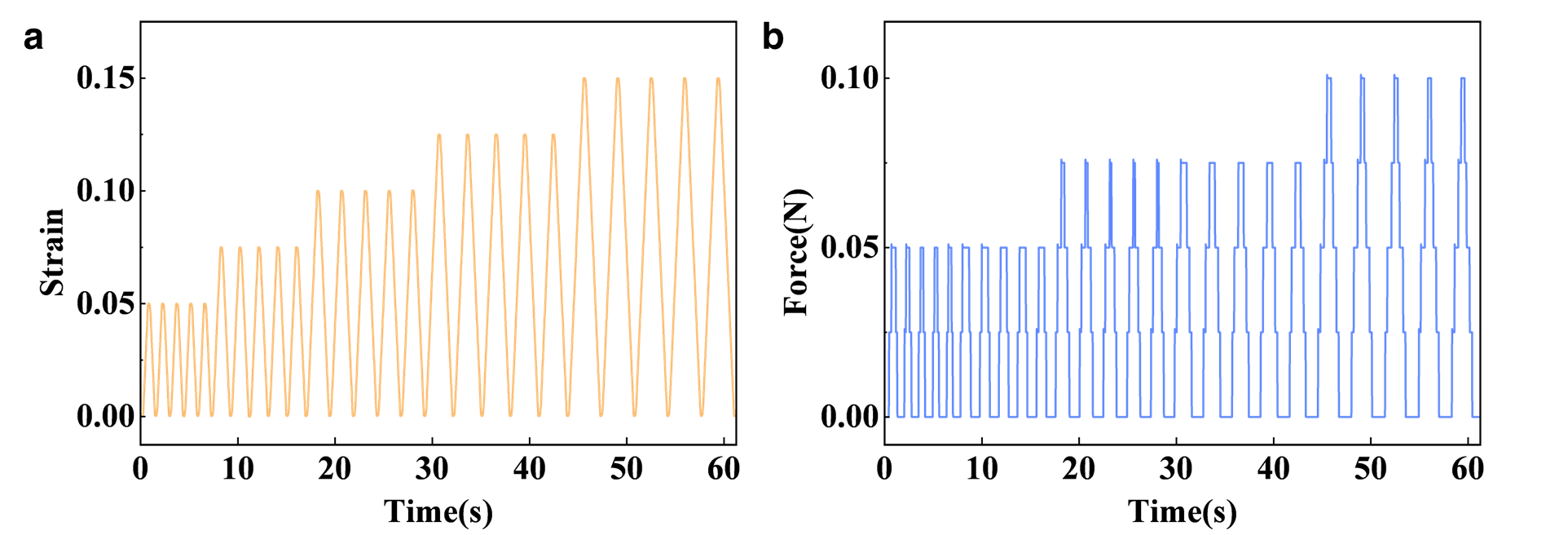
**

**Fig. S14** The diagrams of the mechanical properties. **a** Deformation and **b** tension diagram of the sensor under 5%, 7.5%, 10%, 12.4% and 15% strain

**
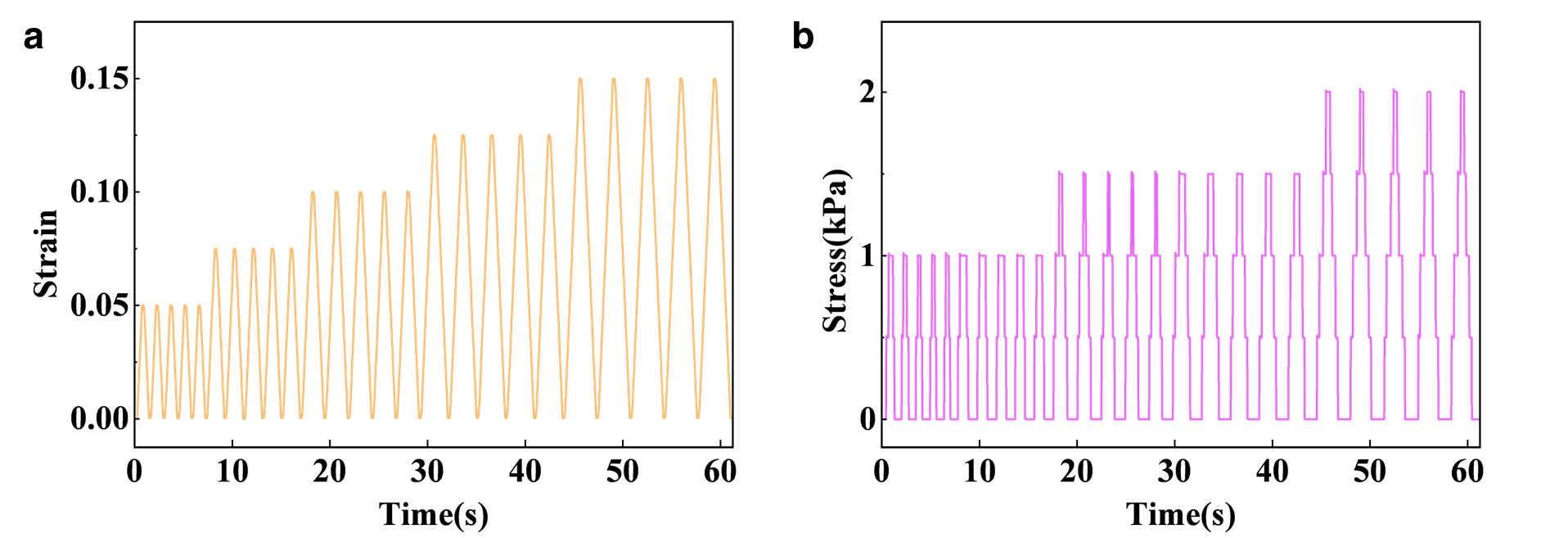
**

**Fig. S15** The diagrams of the mechanical properties. **a** Deformation and **b** stress maps of the sensor under 5%, 7.5%, 10%, 12.4% and 15% strain


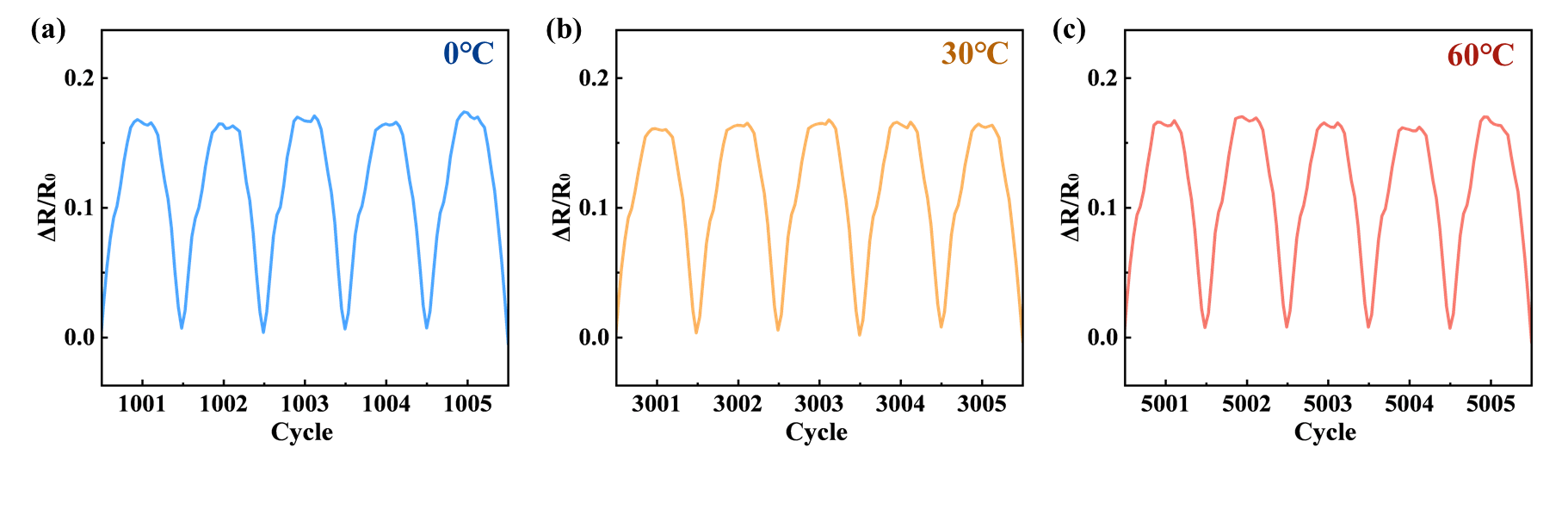


**Fig. S16** Strain resistance curves with representative cyclic periods at different temperatures. **a** cyclic periods at 0 ℃. **b** cyclic periods at 30 ℃. **c** cyclic periods at 60 ℃

**
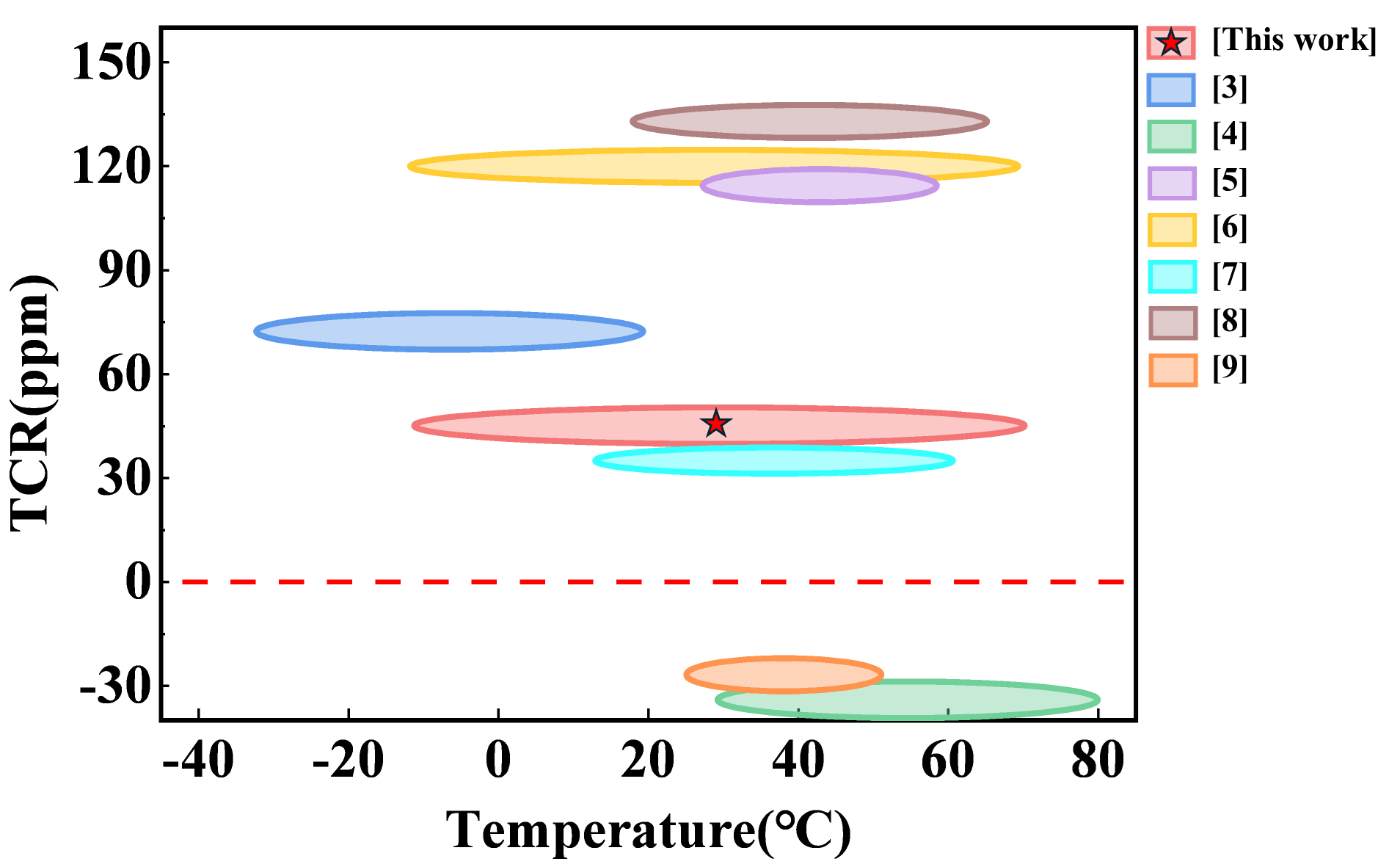
**

**Fig. S17** Comparison of the applicable temperature ranges for other sensors with low resistance coefficient characteristics [S3-S9]

**
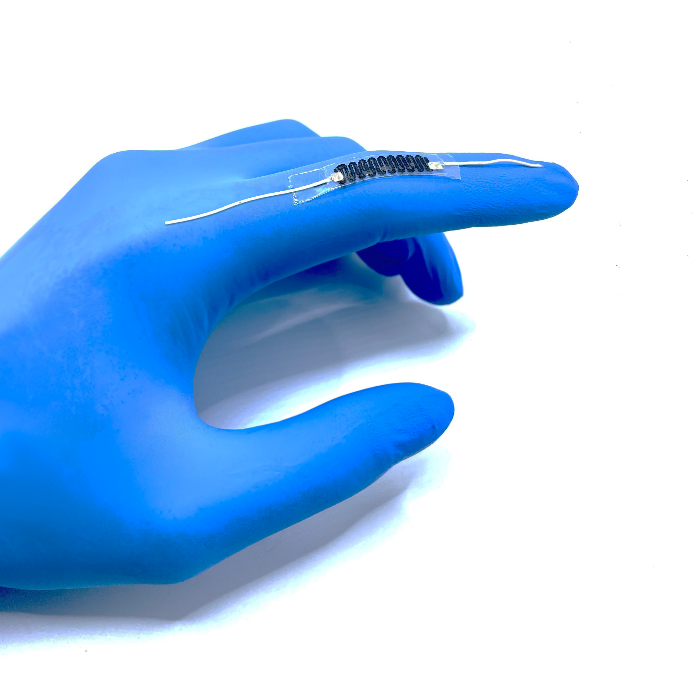
**

**Fig. S18** Schematic of the sensor mounted on the knuckles of the hand


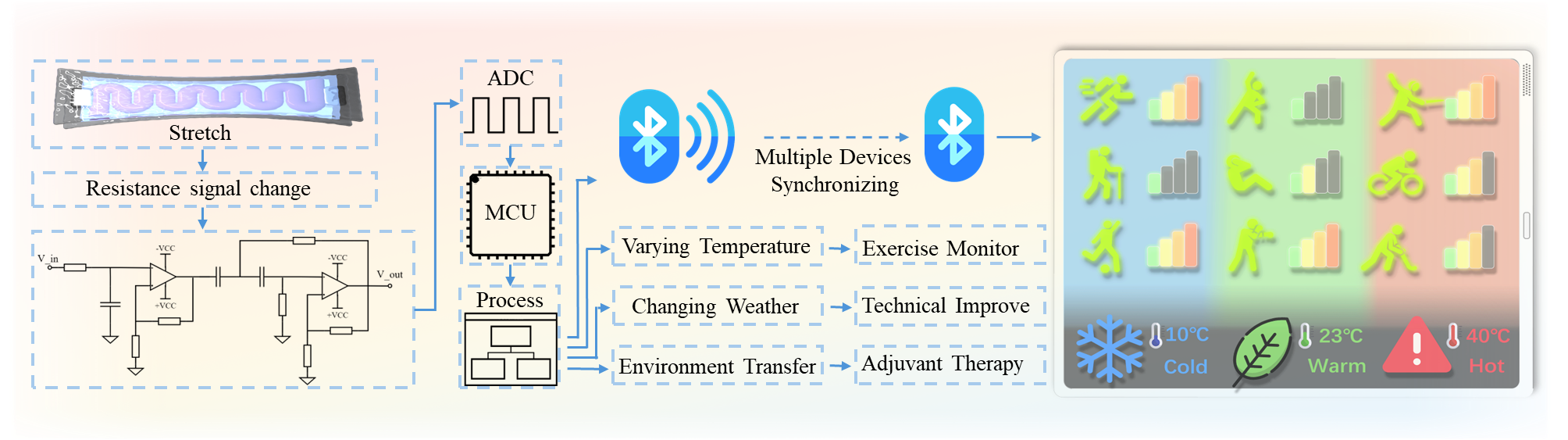


**Fig. S19** System design and multifunctional application diagrams of wearable devices

The electrical signals generated by the strain sensor are processed through filtering, ADC conversion, and MCU handling to obtain real-time strain information. This functionality enables synchronized monitoring of motion across multiple devices under varying temperatures, weather conditions, and environmental changes. Such capability is significant for ensuring reliable data acquisition in complex and dynamic environments, providing robust support for wearable electronics, healthcare monitoring, and intelligent human–machine interaction.

S2 Supplementary Tables

**Table S1** Operating parameter table of electrohydrodynamic direct-writing

| Parameter of  electrohydrodynamic direct writing | Numerical value |
| --- | --- |
| Temperature | 25 ℃ |
| Humidity | 56% RH |
| Applied voltage | 1.76 kV |
| Distance from nozzle to substrate | 1.50 mm |
| Platform movement speed | 60 mm/s |
| Infuse rate | 1 μl/h |
| Outer diameter of needle | 0.23 mm |
| Inner diameter of needle | 0.08 mm |
| Sintering temperature | 300 ℃ |
| Holding time | 60 mins |

**Table S2** Operating parameter table of electrospinning

| Parameter of  electrohydrodynamic direct writing | Numerical value |
| --- | --- |
| Temperature | 25 ℃ |
| Humidity | 56%RH |
| Applied voltage | 18.6 kV |
| Distance from nozzle to substrate | 150 mm |
| Drum speed | 500 rpm |
| Infuse rate | 1000 μl/h |
| Outer diameter of needle | 0.64 mm |
| Inner diameter of needle | 0.34 mm |

The experimental parameters for the electrospinning substrate and the electrohydrodynamic direct writing of high-entropy alloy nanofibers are summarized in the Table. S1 and Table. S2.

**Table S3** Sensor resistance at different temperatures on the variable-temperature stage

| The temperature that  sensor located (℃) | Resistance (kΩ) |
| --- | --- |
| -10 | 353.66 |
| 0 | 353.74 |
| 10 | 353.92 |
| 20 | 354.03 |
| 30 | 354.06 |
| 40 | 354.22 |
| 50 | 354.48 |
| 60 | 354.66 |
| 70 | 354.82 |

The resistance values simultaneously recorded in Table S3 further confirm that the intrinsic resistance variation of the sensor remains minimal across the entire temperature range, demonstrating excellent thermal stability. These direct experimental evidences are fully consistent with the discussion in the main text regarding the sensor’s temperature stability, providing strong and visual support for our core conclusion that “the sensor maintains reliable performance over a wide temperature range.” This supplementary work has significantly enhanced the persuasiveness and completeness of the study.

S3 Supplementary Movie

**Movie S1** Robotics hand grasping and real-time strain signals in different temperatures

Supplementary References

1. W. Zhang, A. Chabok, H. Wang, J. Shen, J. Oliveira et al., Ultra-strong and ductile precipitation-strengthened high entropy alloy with 0.5% Nb addition produced by laser additive manufacturing. J. Mater. Sci. Technol. **187**, 195–211 (2024). <https://doi.org/10.1016/j.jmst.2023.11.053>
2. A. J. de Jesus Silva, M. M. Contreras, C. R. Nascimento, M. F. da Costa, Kinetics of thermal degradation and lifetime study of poly (vinylidene fluoride)(pvdf) subjected to bioethanol fuel accelerated aging. Heliyon. **6**(7), (2020). <https://doi.org/10.1016/j.heliyon.2020.e04573>
3. S. Niu, X. Chang, Z. Zhu, Z. Qin, J. Li et al., Low-temperature wearable strain sensor based on a silver nanowires/graphene composite with a near-zero temperature coefficient of resistance. ACS Appl. Mater. Interfaces. **13**(46), 55307–55318 (2021). <https://doi.org/10.1021/acsami.1c14671>
4. M.-X. Xu, C. Dou, T.-Y. Song, X. Li, Q. Zhang, A temperature-insensitive silver nanostructures@graphene foam for high accuracy and full range human health monitoring. Rare Metals. **43**(11), 5953–5963 (2024). <https://doi.org/10.1007/s12598-024-02758-x>
5. W.-B. Zhu, S.-S. Xue, H. Zhang, Y.-Y. Wang, P. Huang et al., Direct ink writing of a graphene/cnt/silicone composite strain sensor with a near-zero temperature coefficient of resistance. J. Mater. Chem. C. **10**(21), 8226–8233 (2022). <https://doi.org/10.1039/D2TC00918H>
6. J. Ma, Q. Liu, L. Yang, M. Li, Q. Liu et al., A flexible, highly accurate, and stable pressure sensor with anti-interference from temperature, sweat, and humidity. Adv. Mater. Interfaces. **10**(6), 2201528 (2023). <https://doi.org/10.1002/admi.202201528>
7. Y. Zhao, J. Liu, Y. Ying, H. Chen, W. Wang et al.,Temperature self-compensation thin film strain gauges based on nano-SiO_2_/AgNP composites. J. Mater. Chem. C **12**(32), 12491–12498 (2024). <https://doi.org/10.1039/D4TC01645A>
8. J. Lin, Z. Chen, Q. Zhuang, S. Chen, C. Zhu et al., Temperature-immune, wide-range flexible robust pressure sensors for harsh environments. ACS Appl. Mater. Interfaces. **15**(42), 49642–49652 (2023). <https://doi.org/10.1021/acsami.3c10975>
9. T. Yuan, R. Yin, C. Li, Z. Fan, L. Pan, Ti_3_C_2_T_x_ MXene-based all-resistive dual-mode sensor with near-zero temperature coefficient of resistance for crosstalk-free pressure and temperature detections. Chem. Eng. J. **487**, 150396 (2024). <https://doi.org/10.1016/j.cej.2024.150396>
